# Rational design of galactopyranoside-substituted *N*-heterocyclic carbene palladium(ii) complexes. Stable and efficient catalyst for C–C coupling in aqueous media†

**DOI:** 10.1039/d3ra08031e

**Published:** 2024-01-04

**Authors:** Ariana W. Hobsteter, Marcos J. Lo Fiego, Gustavo F. Silbestri

**Affiliations:** a INQUISUR, Departamento de Química, Universidad Nacional del Sur (UNS)-CONICET Av. Alem 1253 B8000CPB Bahía Blanca Argentina ariana.hobsteter@uns.edu.ar marcos.lofiego@uns.edu.ar gsilbestri@uns.edu.ar

## Abstract

Following a rational design, three novel palladium(ii) complexes bearing galactopyranoside-based *N*-heterocyclic carbene ligands have been synthesized *via* transmetalation of the corresponding Ag(i) complexes. Palladium(ii) complexes have been characterized by NMR, FT-IR and elemental analysis. Catalytic studies, using the Stille and Suzuki–Miyaura cross-coupling reactions as model C–C coupling, reveal that the complexes are active and reusable. The best results in terms of TON values were achieved in aqueous medium using either the *in situ* deacetylation of the catalyst or the previously deacetylated catalyst. The catalytic condition using *in situ* deacetylation was more efficient because it avoids an additional deprotection step.

## Abstract 2

Three palladium(ii) complexes bearing galactopyranoside-based NHC ligands have been synthesized. Catalytic studies reveal that the complexes are active and reusable.

## Graphical abstract

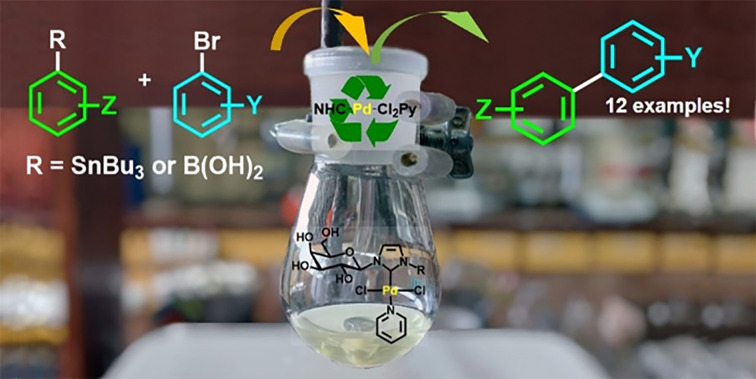

## 1. Introduction

Carbohydrates and their derivatives are important biomolecules with essential physiological functions, *e.g.*, energy production and storage, structural integrity, and biological recognition processes.^1^ Incorporating glycan units into the *N*-heterocyclic carbene (NHC) backbone provides ligands with some unique properties including hydrophilicity, chirality, and biocompatibility.^2^ In addition, their metal complex derivatives are notably attractive as water-soluble catalysts.^2*b*,*d*,3^ However, a limited number of sugar-incorporated NHC complexes with different transition metals have been reported in recent years, holding a variety of bonds between the NHC core and the carbohydrate unit, using generally d-glucose as a representative sugar.^4^ Sugar-incorporated NHC complex catalysts are promising for their low cost and the possibility of replacing organic reaction media with aqueous media in catalysis. The aqueous medium allows easy separation of organic products and their reuse, as well as replacing volatile and flammable organic solvents. Enlargement of these catalysts has been an approach toward environmentally friendly chemical processes.^5^

Palladium-catalyzed cross-coupling reactions are significant methods for carbon–carbon and carbon–heteroatom bond formation. For more than 40 years, the Stille reaction has proven to be an accessible and efficient method for organic synthesis.^6^ The main advantages include the stability and functional group tolerance of stannanes and the broad reaction scope of aryl halides. Therefore, this reaction has increased interest in pharmaceutical and fine chemical industries.^7^ Stille coupling reactions take place in organic solvents and are usually catalyzed homogeneously. Therefore, it is difficult to recover the expensive catalyst from the reaction mixture, leading to waste. Development of a recyclable and reusable catalyst system is valuable in green chemistry terms and practical application. Several strategies have developed involving the use of supported palladium complexes. Besides, implementing green solvents such as water simplifies catalyst separation by creating a biphasic reaction mixture.^8^ In 1990, Casalnuovo *et al.*, reported the Suzuki reaction between aryl halides and boron reagents in water.^9^ This reaction has been crucial in the advancements of aqueous phase cross-coupling reactions.^10^ As a consequence, numerous catalysts capable of promoting Suzuki coupling under mild conditions in pure water or in combination with co-solvents are currently known.^11^

Regarding carbohydrate-based Pd–NHC, in 2007 Glorius *et al.*,^4*b*^ reported the synthesis and characterization of the first Pd(ii)–(NHC)_2_ complex starting from α-d-glucopyranosyl bromide and mesityl imidazole *via* Ag(i)–(NHC)_2_ complex. Later, Lin *et al.*^4*e*^ prepared three different Pd–NHC complexes based on C6-substituted glucopyranose through a five-step sequential approach. The debenzoylated complexes were active and reusable for three cycles in a Suzuki–Miyaura type reaction, in water at 100 °C. On the other hand, Nishioka *et al.*^12^ reported the first carbohydrate-based C–C–N pincer-type Pd–NHC complexes *via* a Click type reaction. Moreover, the same group synthesized a series of similar complexes by changing the terminal alkyl groups (methyl, isopropyl, benzyl, and d-glucopyranosyl) in the NHC moiety.^3*b*^ All complexes were investigated as catalysts in aqueous Suzuki–Miyaura reactions, using K_2_CO_3_ to remove the acetyl groups which improved both solubility and yield. Finally, the same group reported the synthesis of complexes Pd–[bis-NHC-(CH_2_)_2_]Cl_2_ and Pd–[bis-NHC-(CH_2_)_3_]Cl_2_ and their catalytic ability in water.^13^ These complexes exhibited a moderate catalytic activity, decomposing after three uses. The yields improved with the addition of tetrabutylammonium bromide, enhancing the solubility of the aryl halide. Recently, Zhou *et al.*^3*c*^ synthesized and fully characterized glucopyranoside-functionalized *N*-heterocyclic carbenes based pyridine-enhanced precatalyst preparation, stabilization and initiation (PEPPSI) type palladium(ii) complexes and their catalytic activity in Suzuki reaction. The synthesis involves three steps starting from d-glucose to obtain the NHC precursors. They found that a less flexible and bulkier substituent around the palladium metal probably contributed to the formation of isomers mixture. All complexes show good catalytic activity and recyclability in the Suzuki–Miyaura reaction carried out in a solvent mixture of EtOH/H_2_O. Furthermore, the same group reported the synthesis of four Pd–NHC PEPPSI complexes that contain glucose and different alkyl chains (C_*n*_, with *n* = 1, 3, 8 and 16).^3*d*^ The catalyst with the longest alkyl chain proved to be the most efficient in the Suzuki–Miyaura transformation.

Inspired by our previous work on the synthesis and catalytic evaluation of galactopyranoside-incorporated Au(i)–NHC complexes,^14^ we performed a rational design of analogous Pd(ii)–PEPPSI type precatalyst complexes. The new targets were based on d-galactopyranose due to the commercial availability of its precursors and their almost unexplored application in this topic. On the other hand, the substituents on the imidazole nitrogen will allow us to understand correlations between complex structures and catalyst activities. The complexes were fully characterized and evaluated as catalysts in Stille and Suzuki–Miyaura cross-coupling reactions in aqueous medium with excellent results.

## 2. Results and discussion

**Scheme 1:**
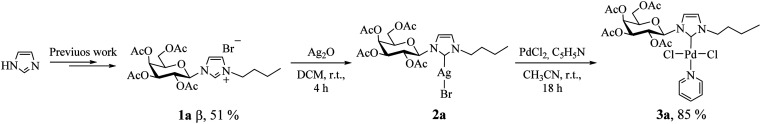
Procedure for synthesis of Pd(ii)–NHC complex 3a.

According to our recent report,^14^ we carried out the synthesis of the imidazolium salt 1a from imidazole, in 51% yield as pure β anomer. Subsequently, we adapted the synthetic strategies reported by Lin *et al.*, to synthesize palladium(ii) complex 3a using 2a as NHC transfer agents.^4*e*^ This method employed PdCl_2_ as the metal precursor and incorporated stoichiometric amounts of pyridine as the fourth coordinating ligand (Scheme 1; see Experimental section for details).

It is noteworthy to mention that, Lee *et al.*^15^ reported the exclusive formation of PdCl_2_Py_2_ using PdCl_2_ as palladium source by attempting to synthesize palladium(ii) complexes using imidazolium salts as precursors and pyridine as both base and solvent. Nevertheless, in our optimized reaction conditions, we obtained PdCl_2_Py_2_ as a byproduct. All the efforts to prevent its formation by modifying reaction variables including temperature and stoichiometry did not produce favorable results. However, it was successfully separated by column chromatography (hexane : ethyl acetate 1 : 1). Another aspect to highlight is that the reaction was carried out at room temperature, while the literature suggests 80 or 100 °C.^3*c*,*d*^ Besides, under the employed reaction conditions formation of bis-carbene complex [Pd(ii)–(NHC)_2_] was not detected. Palladium(ii)–carbene complex 3a was fully characterized by ^1^H, ^13^C NMR, and 2D-HSQC, FT-IR spectroscopy and elemental analysis (see Experimental section for details). The ^1^H NMR data unambiguously confirmed the metal coordination by the disappearance of the proton signal of the imidazole ligand (singlet at *δ* 10.57 ppm). In addition, the ^13^C NMR spectra display the characteristic signal of the carbene carbon bound to palladium with value of 151.5 ppm,^16^ shifted to higher ppm, relative to the starting salts (136.8 ppm). Complex 3a was found to be stable at air and can be stored for prolonged periods.

In order to study the catalytic activity of complex 3a, we assessed the Stille cross-coupling reaction in both organic solvents and aqueous medium. Table 1 summarizes the main results obtained.

**Table 1:** Initial study of Stille cross-coupling reaction^a^

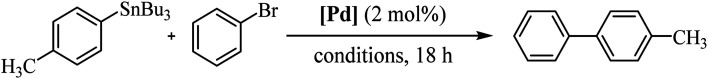					
Entry	Complex	Solvent (1.5 mL)	Additive	Temp (°C)	Yield^b^^,^^c^ (%)
1	3a	Toluene	—	80	27
2	3a	DMF	—	80	63
3^d^	3a	DMF	—	80	69
4	3a	DMF	—	100	60
5^e^	3a	DMF/H_2_O	—	80	36
6^e^	3a	DMF/H_2_O	K_2_CO_3_	80	85^f^
7	3a	H_2_O	K_2_CO_3_	80	38
8^e^	4a	DMF/H_2_O	—	80	87^g^
9	4a	H_2_O	—	80	30

a Relation ArSn/PhBr 1 : 1.2.

b Determinated by GC, with an external standard.

c Homo-coupling product <10–15%.

d 48 h.

e DMF/H_2_O = 1 : 5.

f TON = 43.

g TON = 44.

We initiated the study by performing a cross-coupling reaction between (*p*-methylphenyl)tributylstannane and bromobenzene as a model system. The transformation was conducted in toluene at 80 °C with addition of 2.0 mol% of catalyst. Under these reaction conditions, the desired product was achieved after 18 h in 27% yield (Table 1, entry 1). A second experiment carried out in DMF, a polar solvent suitable for both reagents and catalyst, significantly increased the yield of the reaction to 63% (entry 2). However, a 48-hours reaction did not cause any change in performance (entry 3). Likewise, raising the temperature did not enhance the reaction; instead, it resulted in a decline in the catalytic efficacy (entry 4). Next, we continued the study using DMF/H_2_O (1 : 5) mixture as solvent, at 80 °C. Water was added to the reaction medium for its environmental benefits as well as for its role to facilitate the separation of compounds that are insoluble in water. In these conditions the reaction yield significantly reduced to 36%, probably due to the low catalyst solubility (entry 5). To improve the solubility of the complex in water, we conducted an experiment by adding K_2_CO_3_ to achieve *in situ* deacetylation of the carbohydrate unit. Interestingly, the conversion rate was quantitative (entry 6). Additionally, after a second catalytic cycle, we verified that the catalyst remained active, although its activity decreased significantly to 19% yield (see Experimental section for details of catalyst reuse process).

A similar reaction as in entry 6 using pure water gave the same result as the base-free reaction, indicating that the reactants are insoluble in these conditions (entry 7).

**Scheme 2:**
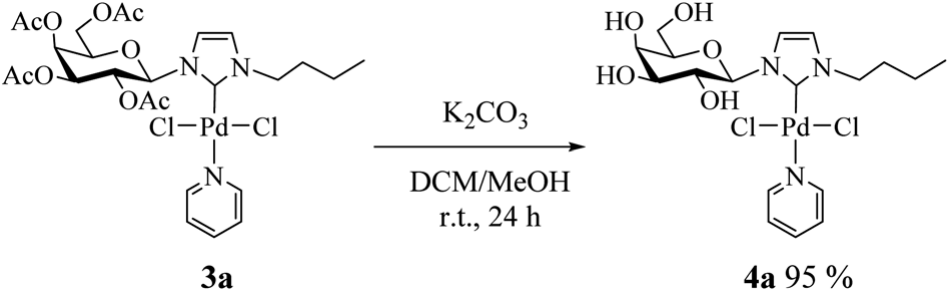
Procedure for preparation of deacetylated Pd(ii)–NHC complex 4a.

Consequently, we decided to isolate the deacetylated complex (4a) and evaluate it as a catalyst. To perform the deacetylation step, the complex 3a was dissolved in DCM/MeOH and K_2_CO_3_ was added.^17^ The reaction mixture was stirred at room temperature for 24 h and after removing the solvent the deacetylated complex was obtained as a yellow solid (Scheme 2, see Experimental section for details). Complex 4a was characterized by NMR noting the disappearance of signals corresponding to acetyl groups.^18^

The catalytic evaluation of 4a, was carried out employing the conditions described in entry 5. This reaction using 4a yielded similar results to those obtained through the *in situ* deprotection conditions (Table 1, compare entries 6 and 8). However, as can be seen in entry 9, the yield decreased significantly when the reaction was performed in pure water, similar to the observations in entry 7.

**Scheme 3:**
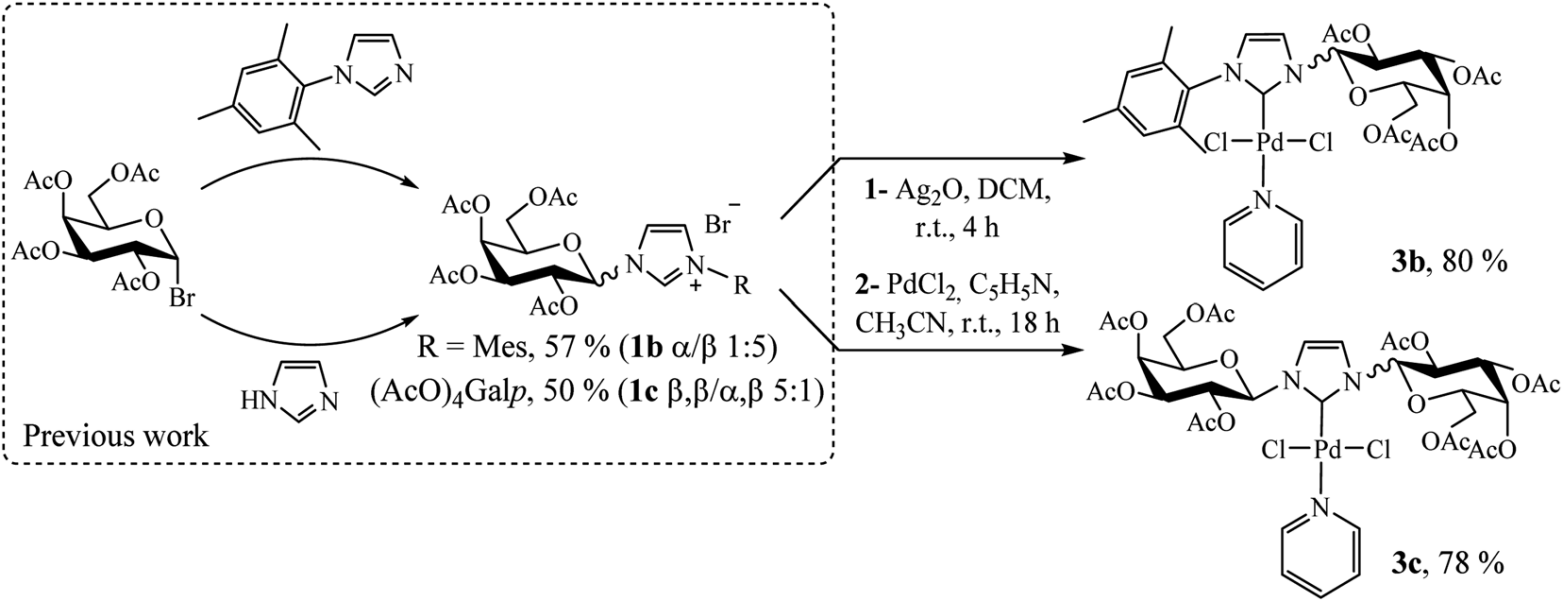
Procedure for preparation of Pd(ii)–NHC complexes 3b and 3c.

Following a rational design, our main goal was to evaluate the impact of substituents on the imidazole nitrogen in Pd(ii)–NHC complexes in terms of stability and catalytic activity. Based on the previously reported bulky imidazolium salts,^14^ we synthesized two novel compounds following a similar procedure employed for complex 3a (Scheme 3, see Experimental section).

The relevant results for 3b and 3c complexes as catalysts in the Stille cross-coupling reaction with K_2_CO_3_, in DMF/H_2_O, at 80 °C are summarized in Table 2. As can be seen, the catalytic activity of both complexes is similar in terms of TON values or CG yield, with a slightly higher activity for complex 3c (entries 1 and 2). Even in an experiment with a reduced loading of 3c (1.0 mol%) no significant changes were observed (entry 3). In contrast, a decrease in catalytic activity was observed with the addition of 0.2 mol% of 3c. However, it is important to highlight that the turnover number increased more than 7 times compared with 2 mol% and more than 3 times compared with 1 mol% (entry 4). In comparison, commercial PdCl_2_ and mixed PdCl_2_/1c (1 : 1) catalyst with 1.0 mol% loading provided 2% and 6% yields, respectively (entries 5 and 6). Furthermore, when PdCl_2_/1c/C_5_H_5_N (1 : 1 : 1) was added, 13% was obtained (entry 7).

**Table 2:** Cross-coupling reactions catalyzed by 3b–c at optimal conditions^a^

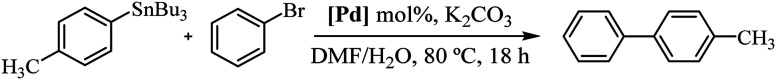			
Entry	Catalyst (mol%)	TON	Yield^b^^,^^c^ (%)
1	3b (2.0)	43	86
2	3c (2.0)	45	90
3	3c (1.0)	87	87
4	3c (0.2)	315	63
5	PdCl_2_ (1.0)		2
6	PdCl_2_/1c (1.0, 1 : 1)		6
7	PdCl_2_/1c/C_5_H_5_N (1.0, 1 : 1 : 1)		13

a Relation ArSn/PhBr 1 : 1.2; DMF/H_2_O (1 : 5).

b Determinated by GC, with an external standard.

c Homo-coupling product <10–15%.

All complexes were stable, but since that 3c showed better performance, we decided to study the reactivity of a set of arylstannanes and aryl bromides with different steric and electronic demands (Table 3).

**Table 3:** Stille reaction catalyzed by 3c^a^

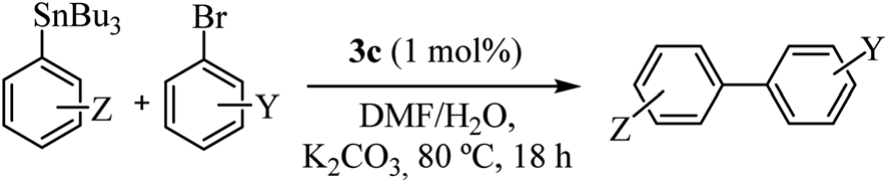			
Entry	Z	Y	Yield^b^^,^^c^ (%)
1	*m*-CH_3_	H	84
2	*m*-Cl	H	68
3	*o*-OCH_3_	H	70
4	*m*-OCH_3_	H	64
5	*p*-OCH_3_	H	77
6	H	*m*-CH_3_	82
7	H	*o*-OCH_3_	76
8	H	*m*-OCH_3_	85
9	H	*p*-OCH_3_	89
10	H	*p*-CN	77

a Relation ArSn/PhBr 1 : 1.2; DMF/H_2_O (1 : 5).

b Determinated by GC, with an external standard.

c Homo-coupling product <10–15%.

As shown in Table 3, the catalyst was found to be active and efficient with all selected reactant pairs. In general, no significant correlations were found between the electronic and steric effects of substituents on the arylstannane and the outcome of the reactions. Nevertheless, the highest yields are achieved when electron-donating groups are present in the haloarene. This study represents the first report of carbohydrate-containing Pd–NHC catalysts in the Stille reaction.

Recognizing the prominence of Suzuki–Miyaura type reactions in forming C–C bonds and their efficiency in an aqueous medium, we aimed to evaluated our catalyst in such transformations. To achieve this, we have replaced the arylstannane with boronic acid and conducted the Suzuki–Miyaura reaction study using complex 3c (Table 4).^19^

**Table 4:** Suzuki–Miyaura cross-coupling catalyzed by 3c in H_2_O^a^

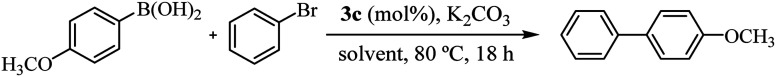				
Entry	3c (mol%)	Solvent	TON	Yield^b^^,^^c^ (%)
1	1.0	H_2_O	73	73
2	1.0	DMF/H_2_O	87	87
3	0.2	DMF/H_2_O	300	60

a Relation ArB(OH)_2_/PhBr 1.5 : 1; DMF/H_2_O (1 : 5).

b Determinated by GC, with an external standard.

c Homo-coupling product <10–15%.

As shown in Table 4, the cross-coupling product was obtained in a 73% yield in pure water (TON = 73). A similar result was found with the tin derivative in DMF/H_2_O (compare with Table 3, entry 5, TON = 77). Notably, when the reaction was carried out in DMF/H_2_O, the yield increased to 87% (TON = 87), possibly due to the improved solubility of bromobenzene in the reaction mixture. Additionally, after a second catalytic cycle, we verified that the catalyst remained active without a significant loss of activity (72% yield). The most important advantage lies on the recyclability and reuse of 3c being reused up to two times in our reaction scale. However, on a larger scale probably it can be reused multiple times. Similar to the reaction carried out with an arylstannane, decreasing the catalyst loading to 0.2 mol% increased the turnover number by more than 3 times (compare entry 3 with entry 4 in Table 2). Complex 3c showed moderate catalytic activity in the Suzuki–Miyaura reaction compared to similar carbohydrate-containing Pd–NHC catalysts tested under similar conditions.^3*c*,*d*^

## 3. Experimental section

### General procedures

The solvents were distilled, dried and stored according to standard procedures.^20^ Unless otherwise stated, reagents were obtained from commercial sources and were used as received. The imidazolium salts were synthesized according to our previously reported method.^14^^1^H, ^13^C NMR, and 2D-HSQC spectra were recorded with a Bruker Advance 300 spectrometer. Chemical shifts (*δ*) are reported in ppm with the residual solvent resonance signal: *δ* H/C 7.27 : 77.2 for CDCl_3_; and *δ* H 4.79 for D_2_O. Melting points were determined on a Reichert–Kofler hot-stage microscope and were uncorrected. Microanalytical data were obtained using an Exeter Analytical Inc. CE-440 microanalyzer. Infrared spectra were collected on an FTIR spectrometer Nicolet Nexus-470. Stille coupling and Suzuki–Miyaura reactions mixture were analyzed by gas–liquid chromatography using a Shimadzu GC-14B instrument equipped with a flame-ionization detector and a HP-5MS column (30 m × 0.25 mm × 0.25 mm), using nitrogen as carrier gas. Mass spectra (EI) were obtained at 70 eV on a Hewlett Packard HP-5890 GC/MS instrument equipped with a HP-5972 selective mass detector.

### General procedure for the synthesis of galactopyranoside-substituted-NHC palladium(ii) complexes (3a–c)

In a 25 mL round-bottom flask, equipped with a nitrogen inlet, was prepared a solution of [Ag(i)–NHC–Br], from the imidazolium salt (0.4 mmol) and silver oxide (0.2 mmol), in dry DCM (4 mL). The reaction mixture was stirred at room temperature for 4 h in dark. The solution was filtered through a pad of Celite and the solvent was removed in vacuum. Then, a [Ag(i)–NHC–Br] solution in 1 mL acetonitrile under N_2_, PdCl_2_ (0.4 mmol) and pyridine (0.4 mmol) was added and the mixture was stirred at room temperature. After 18 h, the solution was filtered through a pad of Celite and the solvent was removed in vacuum. The palladium complex was purified by column chromatography (hexane/ethyl acetate 1 : 1).

### 1-Butyl-3-(2,3,4,6-tetra-*O*-acetyl-β-d-galactopyranosyl)imidazol-2-ylidene palladium(ii) pyridine dichloro (3a)

Pale yellow solid, 85% yield; mp 82–84 °C; *R*_f_ = 0.50 (hexane/ethyl acetate 4 : 6). ^1^H NMR (300 MHz, CDCl_3_) *δ* 8.98 (d, *J* = 4.9 Hz, 2H, H_Py_); 7.78 (t, *J* = 8.4 Hz, 1H, H_Py_); 7.36 (t, 2H, H_Py_); 7.23 (d, *J* = 2.2 Hz, 1H, H4_Imi_); 6.96 (d, *J* = 2.1 Hz, 1H, H5_Imi_); 6.77 (d, *J* = 9.2 Hz, 1H, H1β); 5.63–5.48 (m, 2H, H4,2β); 5.41 (dd, *J* = 10.3, 3.3 Hz, 1H, H3β); 4.87–4.64 (m, 1H, NCH_2_); 4.38–4.16 (m, 3H, H5,6β, NCH_2_); 4.09 (dd, *J* = 10.6, 6.3 Hz, 1H, H6β); 2.19 (s, 3H, CH_3_CO); 2.05 (s, 3H, CH_3_CO); 2.02 (s, 3H, CH_3_CO); 2.00 (s, 5H, CH_3_CO, NCH_2_C*H*_2_); 1.48 (q, *J* = 7.4 Hz, 2H, C*H*_2_CH_3_); 1.01 (t, *J* = 7.3 Hz, 3H, CH_3_). ^13^C NMR (75 MHz, CDCl_3_) *δ* 170.6 (CO); 170.4 (CO); 170.1 (CO); 169.9 (CO); 152.6 (C2_Imi_); 151.5 (CH_Py_); 138.3 (CH_Py_); 124.6 (CH_Py_); 122.5 (C5_Imi_); 119.5 (C4_Imi_); 86.9 (C1); 73.8 (C5); 71.3 (C3); 67.8 (C2); 67.3 (C4); 61.1 (C6); 51.2 (NCH_2_); 32.5 (NCH_2_*C*H_2_); 21.2 (CH_3_CO); 20.8 (CH_3_CO); 20.8 (CH_3_CO); 20.6 (CH_3_CO); 20.0 (*C*H_2_CH_3_); 13.8 (CH_3_). FTIR (neat): 3053.8; 2985.6; 2962.1; 2929.4; 1751.4; 1605.6; 1444.8; 1429.6; 1370.3; 1260.6; 1227.6; 1090.2; 1065.2; 921.7; 896.4; 749.9; 700.7. Elemental analysis calcd for C_26_H_35_Cl_2_N_3_O_9_Pd: C 43.93, H 4.96, N 5.91; found C 43.79, H 4.87, N 5.57.

### 1-Mesityl-3-(2,3,4,6-tetra-*O*-acetyl-β-d-galactopyranosyl)imidazol-2-ylidene palladium(ii) pyridine dichloro (3b)

Yellow solid, 80% yield; mp 122–124 °C; *R*_f_ = 0.62 (hexane/ethyl acetate 4 : 6). ^1^H NMR (300 MHz, CDCl_3_) *δ* 8.77 (d, *J* = 5.0 Hz, 2H, H_Py_); 7.68 (t, *J* = 7.6 Hz, 1H, H_Py_), 7.44 (d, *J* = 1.9 Hz, 1H, H4_Imi_); 7.28–7.21 (m, 2H, H_Py_); 7.03 (s, 1H, H_Ar_); 6.98 (d, *J* = 9.2 Hz, 1H, H1β); 6.95 (s, 1H, H_Ar_); 6.90 (d, *J* = 2.0 Hz, 1H, H5_Imi_); 5.67–5.53 (m, 2H, H4,2β); 5.47 (dd, *J* = 10.3, 3.2 Hz, 1H, H3β); 4.43–4.08 (m, 3H, H5,6β); 2.38 (s, 3H, CH_3Ar_), 2.34 (s, 3H, CH_3_CO), 2.22 (s, 3H, CH_3_CO), 2.12 (s, 3H, CH_3_CO), 2.07 (s, 3H, CH_3_CO), 2.04 (s, 3H, CH_3Ar_), 2.03 (s, 3H, CH_3Ar_). ^13^C NMR (75 MHz, CDCl_3_) *δ* 170.6 (CO); 170.5 (CO); 170.2 (CO); 169.9 (CO); 154.3 (C2_Imi_); 151.6 (CH_Py_); 139.4 (C_Ar_); 138.3 (CH_Py_); 136.3 (C_Ar_); 135.9 (C_Ar_); 134.8 (CH_Ar_); 129.3 (CH_Ar_); 125.2 (C5_Imi_); 124.4 (CH_Py_); 119.8 (C4_Imi_); 87.2 (C1); 73.9 (C5); 71.2 (C3); 68.2 (C2); 67.3 (C4); 61.0 (C6); 21.3 (CH_3Ar_); 21.2 (CH_3_CO); 20.9 (CH_3_CO); 20.8 (CH_3_CO); 20.7 (CH_3_CO); 19.3 (CH_3Ar_); 18.6 (CH_3Ar_). FTIR (neat): 3054.0; 2986.8; 1752.6; 1422.3; 1370.6; 1262.7; 1224.4; 896.2; 749.7; 703.5. Elemental analysis calcd for C_31_H_37_Cl_2_N_3_O_9_Pd: C 48.17, H 4.83, N 5.44; found C 47.95, H 4.91, N 4.99.

### 1-(2,3,4,6-Tetra-*O*-acetyl-β-d-galactopyranosyl)-3-(2,3,4,6-tetra-*O*-acetyl-β-d-galactopyranosyl)imidazol-2-ylidene palladium(ii) pyridine dichloro (3c)

Pale yellow solid, 78% yield; mp 102–124 °C; *R*_f_ = 0.40 (hexane/ethyl acetate 1 : 1). ^1^H NMR (300 MHz, CDCl_3_) *δ* 8.98 (d, *J* = 5.1 Hz, 2H, H_Py_); 7.80 (t, *J* = 7.6 Hz, 1H, H_Py_); 7.39 (t, *J* = 6.9 Hz, 2H, H_Py_); 7.26 (s, 2H, H4,5_Imi_); 6.76 (d, *J* = 9.2 Hz, 2H, H1β); 5.59–5.46 (m, 4H, H4,2β); 5.40 (dd, *J* = 10.2, 3.2 Hz, 2H, H3β); 4.33–4.02 (m, 6H, H5,6β); 2.20 (s, 6H, CH_3_CO); 2.04 (s, 6H, CH_3_CO); 1.99 (s, 12H, 2 × CH_3_CO). ^13^C NMR (75 MHz, CDCl_3_) *δ* 170.5 (CO); 170.3 (CO); 170.2 (CO); 169.9 (CO); 156.5 (C2_Imi_); 151.7 (CH_Py_); 138.4 (CH_Py_); 124.7 (CH_Py_); 120.3 (C4,5_Imi_); 87.2 (C1); 74.0 (C5); 71.2 (C3); 67.9 (C2); 67.3 (C4); 61.1 (C6); 21.0 (CH_3_CO); 20.8 (CH_3_CO); 20.7 (CH_3_CO); 20.6 (CH_3_CO). FTIR (neat): 3054.5; 2985.0; 1746.2; 1437.5; 1368.0; 1263.8; 1221.3; 1094.0; 1063.1; 742.8; 704.2. Elemental analysis calcd for C_36_H_45_Cl_2_N_3_O_18_Pd: C 43.89, H 4.60, N 4.27; found C 43.40, H 4.61, N 4.12.

### Procedure for the synthesis of deacetylated complex (4a)

In a 10 mL round bottom flask, 0.1 mmol of 3a was dissolved in 3 mL of DCM/MeOH (1 : 1) and 0.03 mmol of K_2_CO_3_ was added. The reaction mixture was stirred at room temperature for 24 h. The mixture was filtered through a pad of Celite and concentrated in vacuum to give the desired deprotected complex.

### 1-Butyl-3-(β-d-galactopyranosyl)imidazol-2-ylidene palladium(ii) pyridine dichloro (4a)

Yellow solid, 95% yield. ^1^H NMR (300 MHz, D_2_O) *δ* 8.87 (d, *J* = 5.4 Hz, 2H, H_Py_); 7.81 (t, *J* = 7.6 Hz, 1H, H_Py_); 7.34 (s, 1H, H4_Imi_); 7.27 (s, 1H, H5_Imi_); 7.22 (t, 2H, H_Py_); 5.10 (d, *J* = 9.4 Hz, 1H, H1β); 4.79 (s, 2H, NCH_2_); 4.15–4.08 (m, 2H, H3,2β); 4.09–4.00 (m, 1H, H4β); 3.87–3.76 (m, 2H, H6β); 3.48 (dd, *J* = 8.7, 4.0 Hz, 1H, H5β); 1.99–1.86 (m, 2H, NCH_2_C*H*_2_); 1.63–1.52 (m, 2H, C*H*_2_CH_3_); 1.04 (t, *J* = 7.3 Hz, 3H, CH_3_). ^13^C NMR (75 MHz, D_2_O) *δ* 153.2 (C2_Imi_); 139.0 (CH_Py_); 126.0 (C5_Imi_); 125.1 (CH_Py_); 121.4 (C4_Imi_); 89.4 (C1); 86.0 (C5); 78.0 (C3); 77.2 (C2); 72.3 (C4); 62.3 (C6); 52.0 (NCH_2_); 32.2 (NCH_2_*C*H_2_); 19.5 (*C*H_2_CH3), 13.1 (CH_3_).

### General procedure for the Stille cross-coupling reaction catalyzed by palladium(ii) complexes (3a–c)

In a Schlenk tube loaded with 1.5 mL of DMF/H_2_O (1 : 5), 2 mol% of palladium(ii) complex and 0.007 mmol K_2_CO_3_ were added. After stirring for 5 min, 0.48 mmol of bromobenzene and 0.4 mmol of arylstannane were added consequently. The mixture was stirred for 18 h at 80 °C and then ethyl ether (10 mL × 2) was added and extracted. The organic layer was dried over MgSO_4_ and injected on GC-MS to determine to reaction conversion. The product was purified by flash chromatography on silica gel doped with 10% of KF (hexane : EtOAc, 9 : 1–7 : 3). To the aqueous phase containing hydrophilic catalyst, bromobenzene and arylstannane were added. The mixture was stirred at room temperature for 18 h. This process was repeated several times until the transformation was no longer efficient.

### C–C coupling product characterization

#### *o*-Methoxybiphenyl

Colorless oil; ^1^H NMR (300 MHz, CDCl_3_) *δ* 7.60–7.57 (m, 2H), 7.46 (t, *J* = 7.5 Hz, 2H), 7.40–7.33 (m, 3H), 7.13–6.98 (m, 2H), 3.85 (s, 3H); ^13^C NMR (75 MHz, CDCl_3_) *δ* 156.6, 138.6, 131.0, 130.8, 129.7, 128.7, 128.1, 127.0, 120.9, 111.3, 55.7. MS (*m*/*z*, relative intensity): 184 (75, M^+^), 141 (70); 139 (30), 115 (100).

#### *m*-Methoxybiphenyl

Colorless oil; ^1^H NMR (300 MHz, CDCl_3_) *δ* 7.50–7.43 (m, 2H), 7.34–7.26 (m, 2H), 7.25–7.17 (m, 2H), 7.10–6.99 (m, 2H); 6.77 (m, 1H), 3.71 (s, 3H); ^13^C NMR (75 MHz, CDCl_3_) *δ* 160.1, 142.9, 141.2, 129.8 × 2, 127.5, 127.3, 119.8, 113.1, 112.8, 54.4. MS (*m*/*z*, relative intensity): 184 (70, M^+^), 154 (32, M^+^ − 30), 141 (66), 139 (36), 115 (100).

#### *p*-Methoxybiphenyl

White solid; mp 89–91 °C; ^1^H NMR (300 MHz, CDCl_3_) *δ* 7.59–7.52 (m, 4H), 7.47–7.39 (m, 2H), 7.35–7.26 (m, 1H), 6.99 (d, *J* = 8.8 Hz, 2H); 3.86 (s, 3H); ^13^C NMR (75 MHz, CDCl_3_) *δ* 159.4, 141.0, 134.0, 128.9, 128.3, 126.9, 126.8, 114.4, 55.5. MS (*m*/*z*, relative intensity): 184 (83, M^+^), 169 (51, M^+^ − 15), 141 (98), 115 (100).

#### *m*-Methylbiphenyl

Colorless oil; ^1^H NMR (300 MHz, CDCl_3_) *δ* 7.65 (d, *J* = 8.1 Hz, 2H), 7.52–7.43 (m, 4H), 7.42–7.36 (m, 2H), 7.23 (d, *J* = 7.5 Hz, 1H), 2.48 (s, 3H); ^13^C NMR (75 MHz, CDCl_3_) *δ* 141.5, 141.4, 138.4, 128.8 × 2, 128.1 × 2, 127.3 × 2, 124.4, 21.7. MS (*m*/*z*, relative intensity): 168 (100, M^+^); 167 (57, M^+^ − 1); 152 (36); 139 (20); 115 (32).

#### *p*-Methylbiphenyl

Colorless syrup; ^1^H NMR (300 MHz, CDCl_3_) *δ* 7.62 (d, *J* = 7.2 Hz, 2H), 7.54 (d, *J* = 7.9 Hz, 2H), 7.47 (t, *J* = 7.6 Hz, 2H), 7.36 (m, 1H), 7.29 (d, *J* = 7.8 Hz, 2H), 2.44 (s, 3H); ^13^C NMR (75 MHz, CDCl_3_) *δ* 141.3, 138.5, 137.2, 129.6, 128.8, 127.1 × 2, 21.3. MS (*m*/*z*, relative intensity): 168 (100, M^+^), 167 (71, M^+^ − 1), 165 (27), 152 (23).

#### *m*-Chlorobiphenyl

Colorless oil; ^1^H NMR (300 MHz, CDCl_3_) *δ* 7.49–7.42 (m, 3H), 7.38–7.30 (m, 3H), 7.29–7.21 (m, 3H); ^13^C NMR (75 MHz, CDCl_3_) *δ* 143.2, 139.9, 134.8, 130.1, 129.0, 128.0, 127.4, 127.3, 127.2, 125.4. MS (*m*/*z*, relative intensity): 188/190 (3/1, 100, M^+^); 152 (95, M^+^ − Cl); 151 (34, M^+^ − HCl); 126 (20).

#### *p*-Cyanobiphenyl

Colorless syrup; ^1^H NMR (300 MHz, CDCl_3_) *δ* 7.76–7.76 (m, 4H), 7.59 (d, *J* = 7.5 Hz, 2H), 7.53–7.40 (m, 3H); ^13^C NMR (75 MHz, CDCl_3_) *δ* 145.9, 139.3, 132.7, 129.2, 128.8, 127.9, 127.4, 119.2, 111.0. MS (*m*/*z*, relative intensity): 180 (15, M^+^ + 1), 179 (100, M^+^), 178 (27), 177 (11), 152 (9), 151 (17).

## 4. Conclusions

Galactopyranosyl imidazolium salts from aryl- or β-d-galactosyl imidazole as precursors were used to synthesize three new stable Pd(ii)–PEPPSI type precatalyst complexes, in excellent yield under mild conditions, employing PdCl_2_ as metal precursor and stoichiometric amounts of pyridine.

Catalytic studies indicate that the complexes are active and reusable, in the C–C cross-coupling reactions in aqueous medium. It is important to note that the Stille reaction necessarily requires the use of organic solvents to dissolve the reactants. Excellent yields have been obtained using a mixture of DMF/H_2_O, reducing the use of organic solvent by up to 80%. On the other hand, in the Suzuki–Miyaura reaction, excellent yields were observed using pure water or DMF/H_2_O due to the solubility of boronic acid. In both C–C cross-coupling type reactions, a decrease in catalytic activity and an increase of more than 3 times in the turnover number were observed with 0.2 mol% of catalyst. These methodologies allow for excellent conversion percentages and provide a more ecological perspective to these types of transformations.

Further work is under development in our laboratory focused on the effectiveness of these Pd(ii)–NHC complexes in other aqueous phase reactions.

## Author contributions

Ariana W. Hobsteter: methodology, validation, formal analysis, investigation, writing original draft. Marcos J. Lo Fiego: conceptualization, methodology, validation, supervision, formal analysis, writing original draft, writing – review & editing. Gustavo F. Silbestri: conceptualization, funding acquisition, investigation, methodology, project administration, resources, visualization, writing original draft, writing – review & editing. All authors have given approval to the final version of the manuscript.

## Conflicts of interest

There are no conflicts to declare.

## Supplementary Material

RA-014-D3RA08031E-s001
