# Supplementary material for: Rational design of galactopyranoside-substituted N-heterocyclic carbene palladium(ii) complexes. Stable and efficient catalyst for C–C coupling in aqueous media
Source: RSC Adv. 2024 Jan 4;14(3):1626–33. doi: 10.1039/d3ra08031e (PMC10765771; doi:10.1039/d3ra08031e)

Electronic Supplementary Material (ESI) for RSC Advances.
This journal is © The Royal Society of Chemistry 2023

Rational design of Galactopyranoside-substituted *N*-heterocyclic carbene Palladium(II) Complexes. Stable and Efficient catalyst for C-C coupling in aqueous medium

Ariana W. Hobsteter, Marcos J. Lo Fiego[†,*] and Gustavo F. Silbestri[†,*]

INQUISUR, Departamento de Química, Universidad Nacional del Sur (UNS)-CONICET, Av. Alem 1253, B8000CPB Bahía Blanca, Argentina.

* Corresponding authors: marcos.lofiego@uns.edu.ar (M. J. Lo Fiego); gsilbestri@uns.edu.ar (G. F. Silbestri)

† Member of CONICET

Supplementary Material

Table of Contents

^{1}H and ^{13}C NMR of **3a** S2

HSQC and FTIR-neat of **3a** S3

^{1}H and ^{13}C NMR of **3b** S4

HSQC and FTIR-neat of **3b** S5

^{1}H and ^{13}C NMR of **3c** S6

HSQC and FTIR-neat of **3c** S7

^{1}H and ^{13}C NMR of **4a** S8

HSQC of **4a** S9

^{1}H and ^{13}C NMR of ***o*-methoxybiphenyl** S10

^{1}H and ^{13}C NMR of ***m*-methoxybiphenyl** S11

^{1}H and ^{13}C NMR of ***p*-methoxybiphenyl** S12

^{1}H and ^{13}C NMR of ***m*-methylbiphenyl** S13

^{1}H and ^{13}C NMR of ***p*-methylbiphenyl** S14

^{1}H and ^{13}C NMR of ***m*-chlorobiphenyl** S15

^{1}H and ^{13}C NMR of ***p*-cyanobiphenyl** S16

1-Butyl-3-(2,3,4,6-tetra-*O*-acetyl-β-D-galactopyranosyl)imidazol-2-ylidene palladium(II) pyridine dichloro (**3a**, ^{1}H NMR, $CDCl_3$)

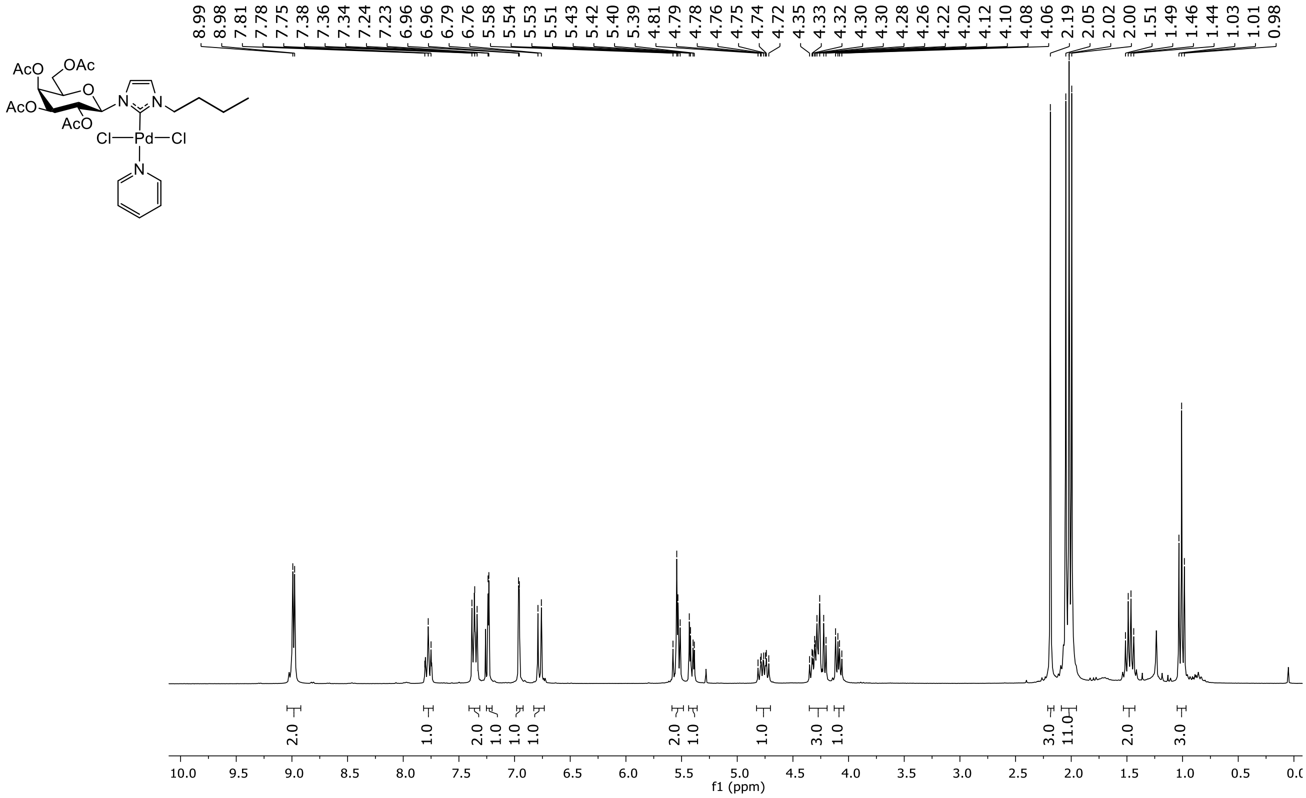

1-Butyl-3-(2,3,4,6-tetra-*O*-acetyl-β-D-galactopyranosyl)imidazol-2-ylidene palladium(II) pyridine dichloro (**3a**, ^{13}C NMR, $CDCl_3$)

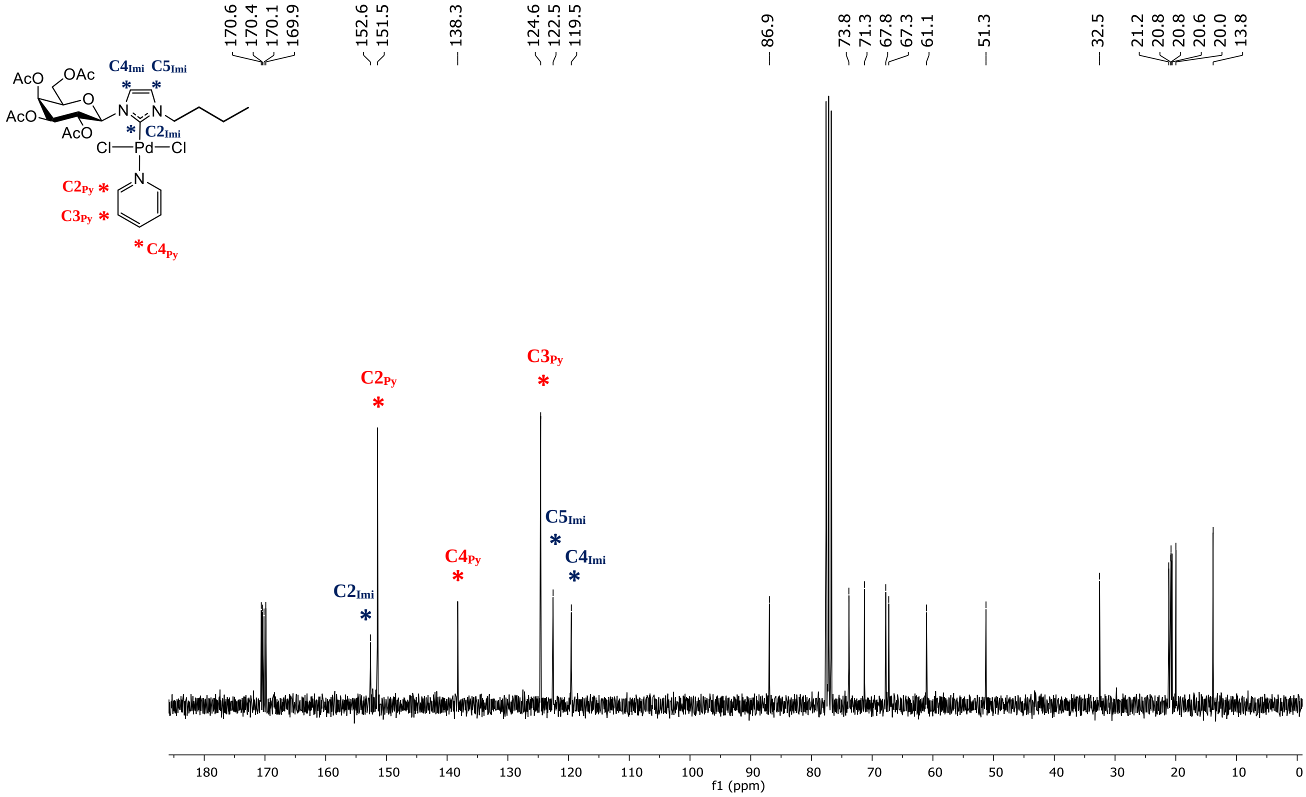

1-Butyl-3-(2,3,4,6-tetra-*O*-acetyl-β-D-galactopyranosyl)imidazol-2-ylidene palladium(II) pyridine dichloro (**3a**, HSQC, $CDCl_3$)

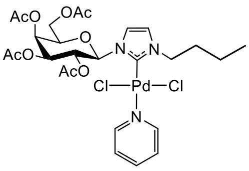

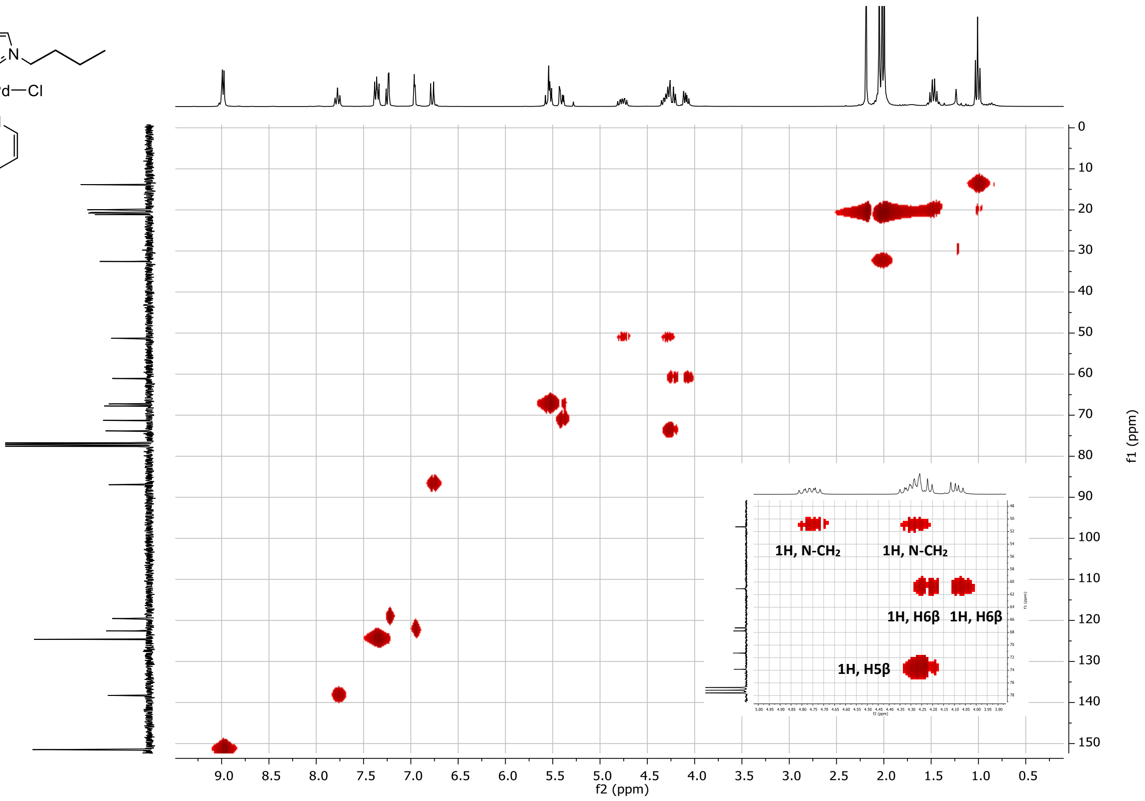

1-Butyl-3-(2,3,4,6-tetra-*O*-acetyl-β-D-galactopyranosyl)imidazol-2-ylidene palladium(II) pyridine dichloro (**3a**, FTIR-neat)

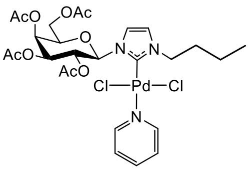

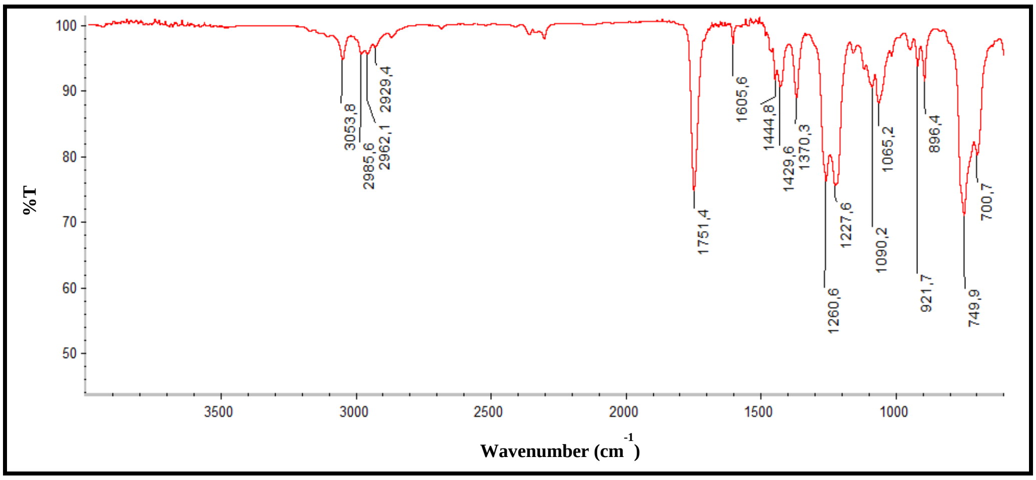

1-Mesityl-3-(2,3,4,6-tetra-*O*-acetyl-β-D-galactopyranosyl)imidazol-2-ylidene palladium(II) pyridine dichloro (**3b**, ^{1}H NMR, $CDCl_3$)

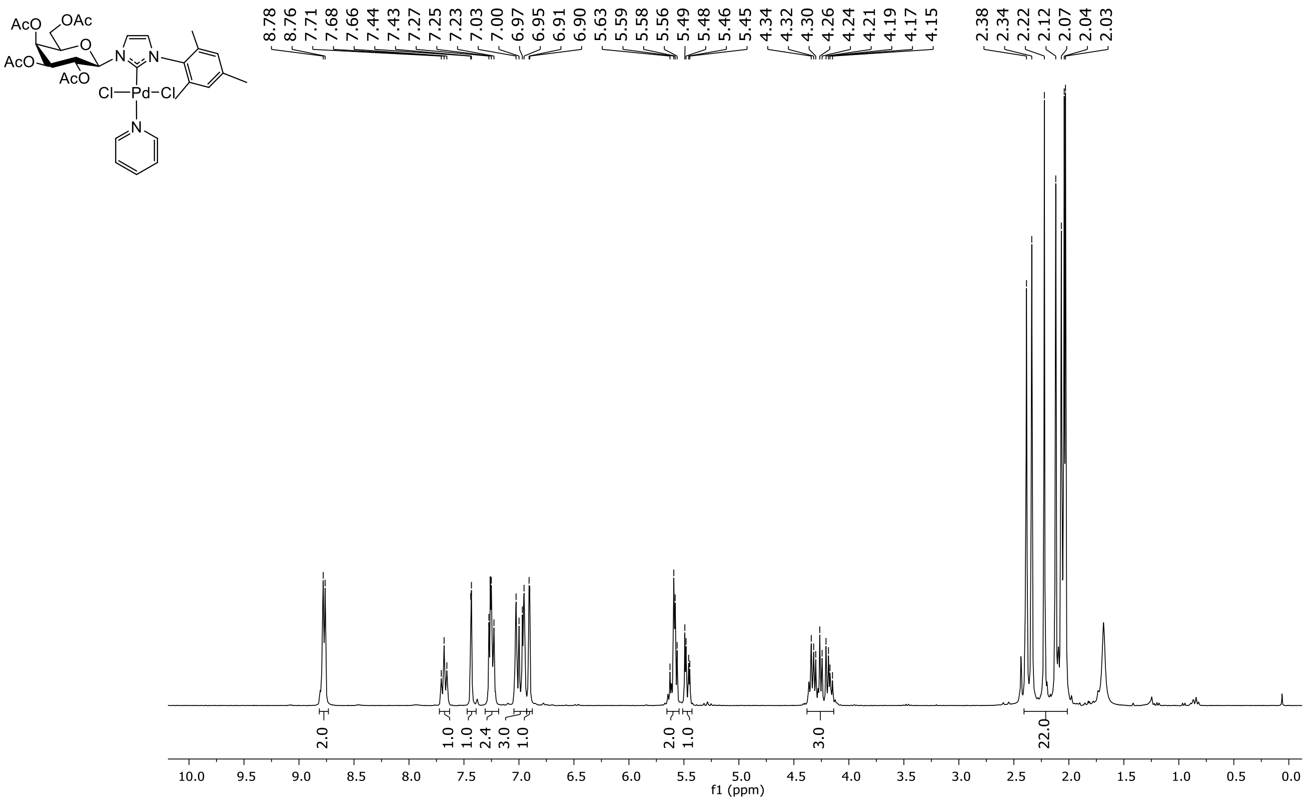

1-Mesityl-3-(2,3,4,6-tetra-*O*-acetyl-β-D-galactopyranosyl)imidazol-2-ylidene palladium(II) pyridine dichloro (**3b**, ^{13}C NMR, $CDCl_3$)

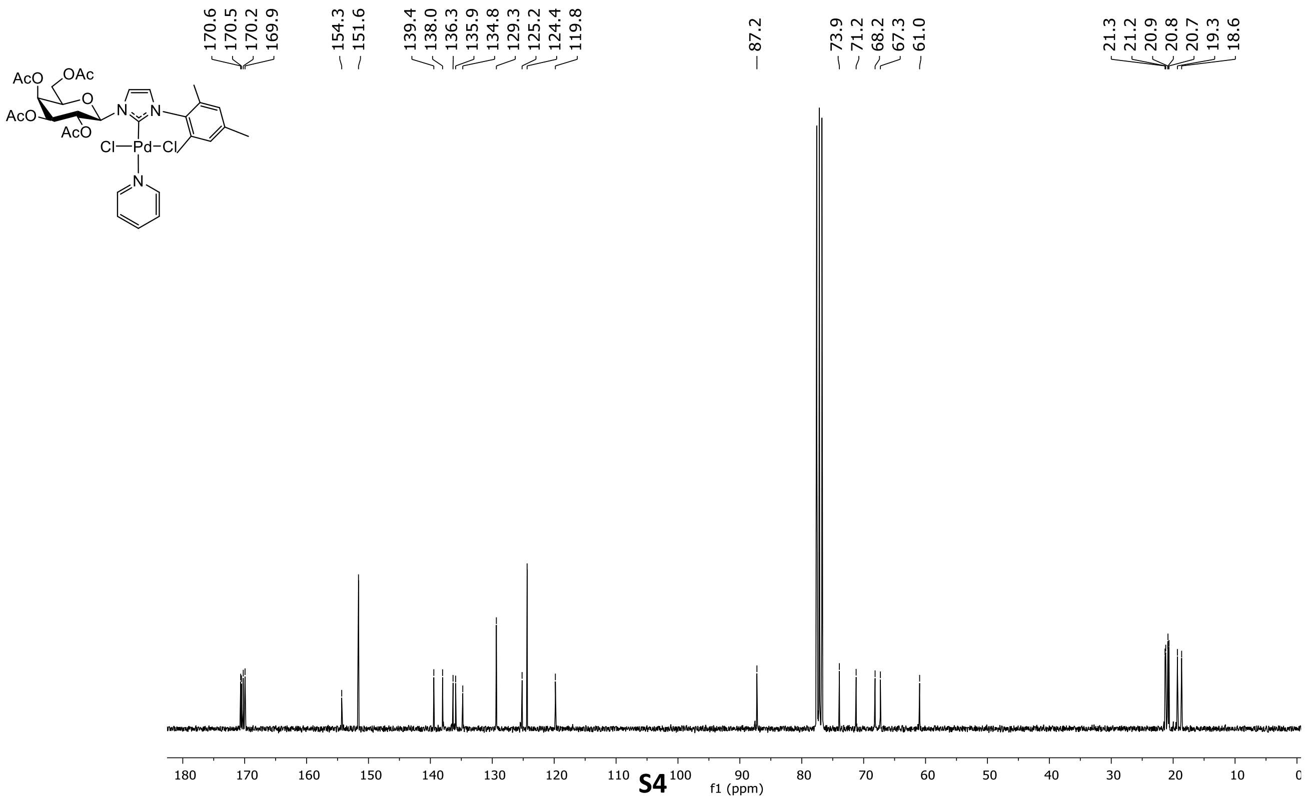

1-Mesityl-3-(2,3,4,6-tetra-*O*-acetyl-β-D-galactopyranosyl)imidazol-2-ylidene palladium(II) pyridine dichloro (**3b**, HSQC, $CDCl_3$)

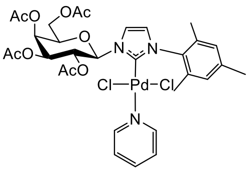

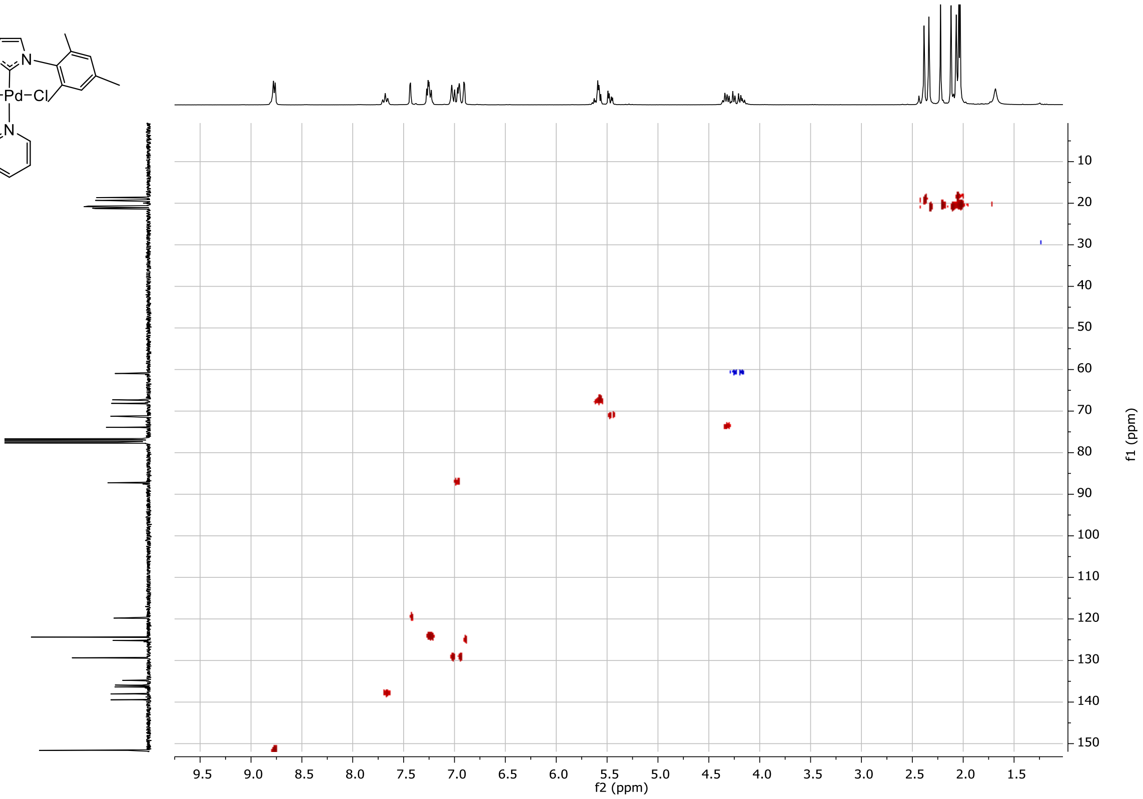

1-Mesityl-3-(2,3,4,6-tetra-*O*-acetyl-β-D-galactopyranosyl)imidazol-2-ylidene palladium(II) pyridine dichloro (**3b**, FTIR-neat)

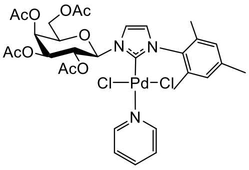

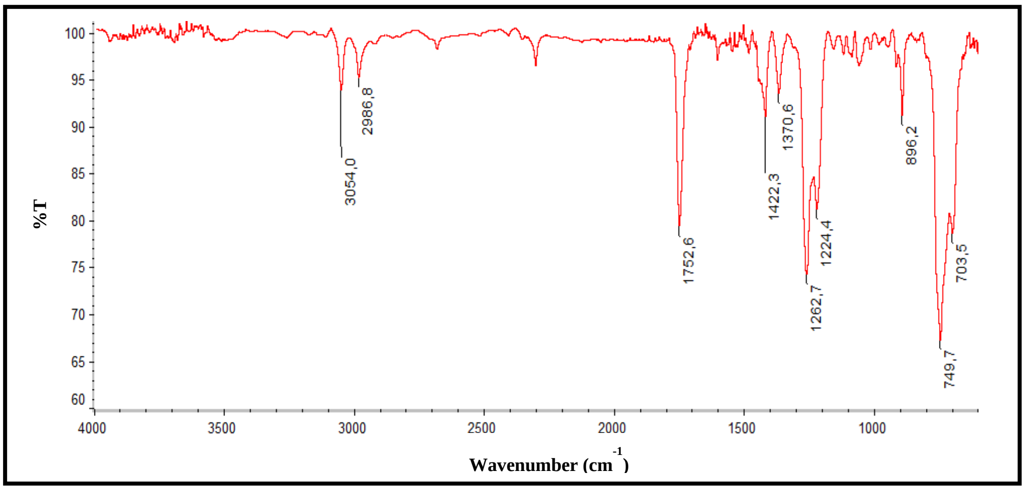

1-(2,3,4,6-Tetra-*O*-acetyl-β-D-galactopyranosyl)-3-(2,3,4,6-tetra-*O*-acetyl-β-D-galactopyranosyl) imidazol-2-ylidene palladium(II) pyridine dichloro (**3c**, ^{1}H NMR, $CDCl_3$)

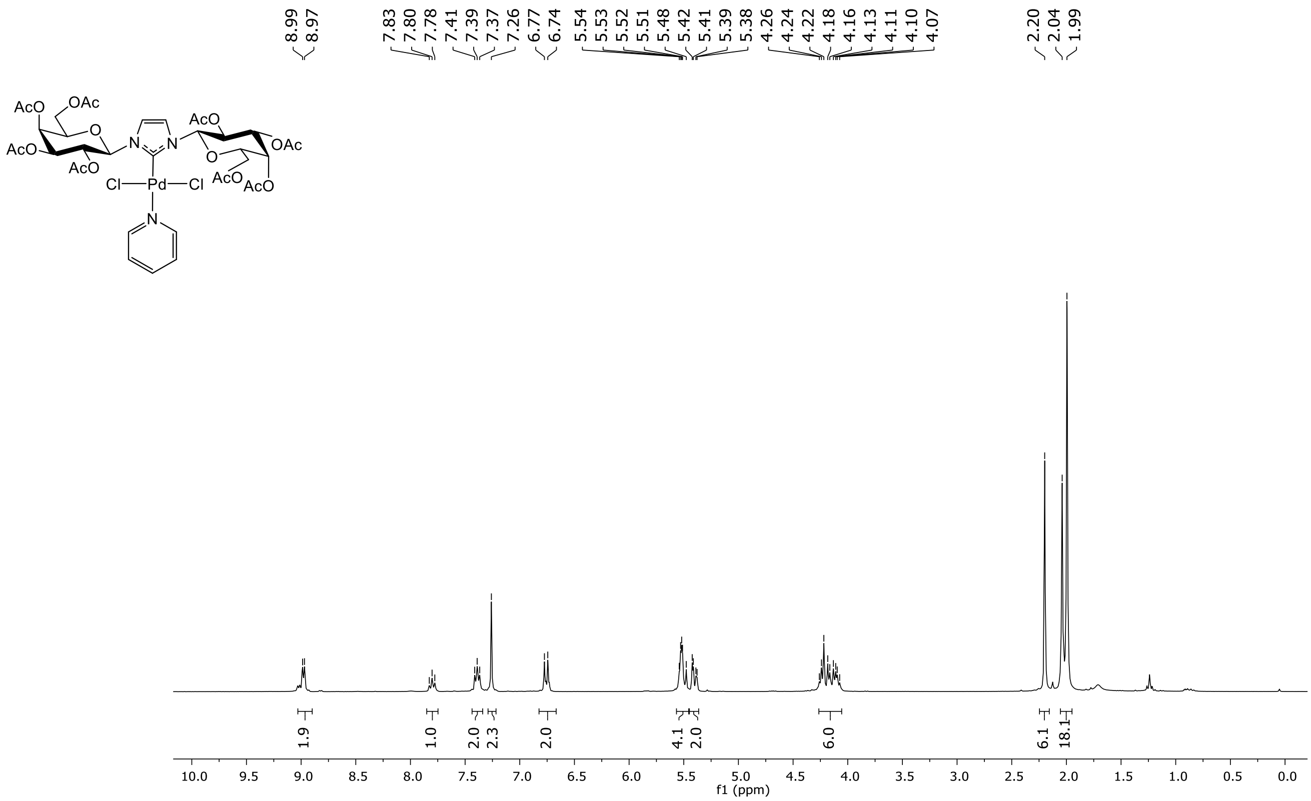

1-(2,3,4,6-Tetra-*O*-acetyl-β-D-galactopyranosyl)-3-(2,3,4,6-tetra-*O*-acetyl-β-D-galactopyranosyl) imidazol-2-ylidene palladium(II) pyridine dichloro (**3c**, ^{13}C NMR, $CDCl_3$)

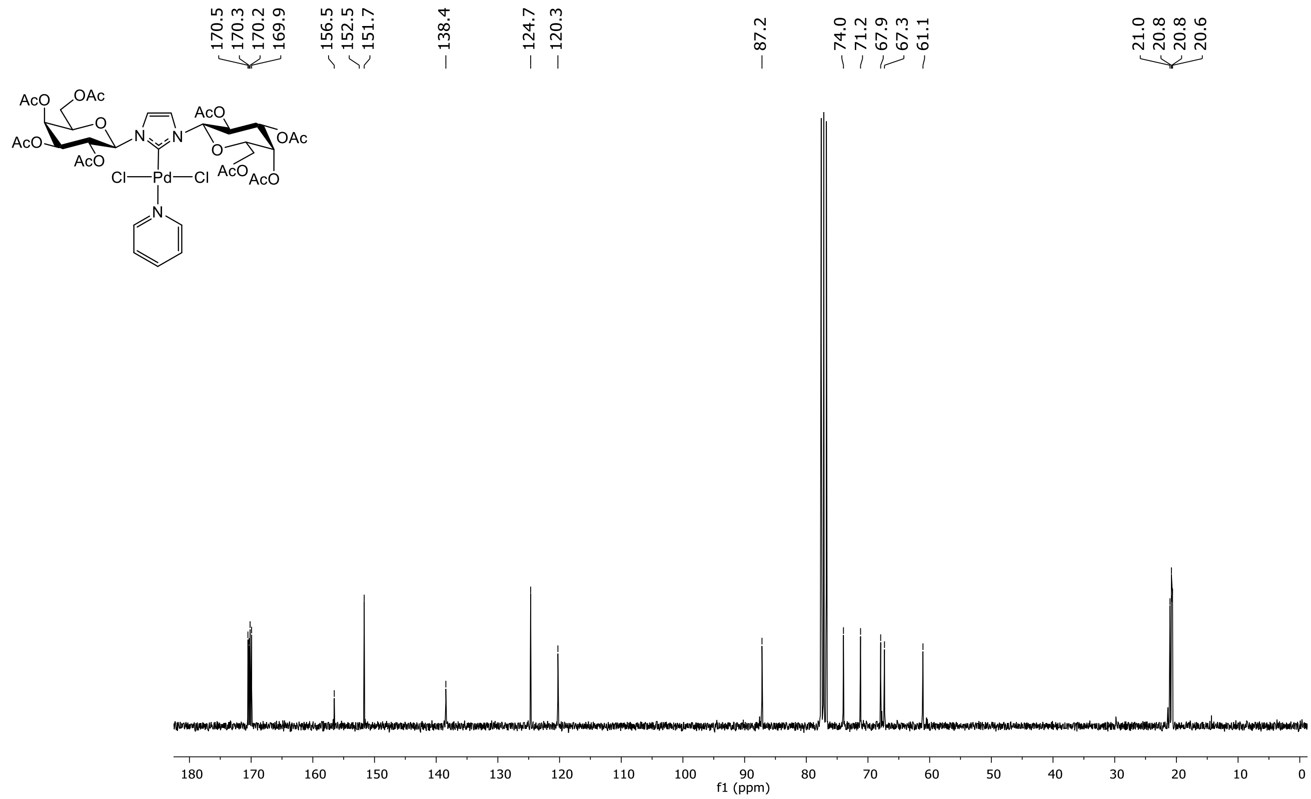

1-(2,3,4,6-Tetra-*O*-acetyl-β-D-galactopyranosyl)-3-(2,3,4,6-tetra-*O*-acetyl-β-D-galactopyranosyl) imidazol-2-ylidene palladium(II) pyridine dichloro (**3c**, HSQC, $CDCl_3$)

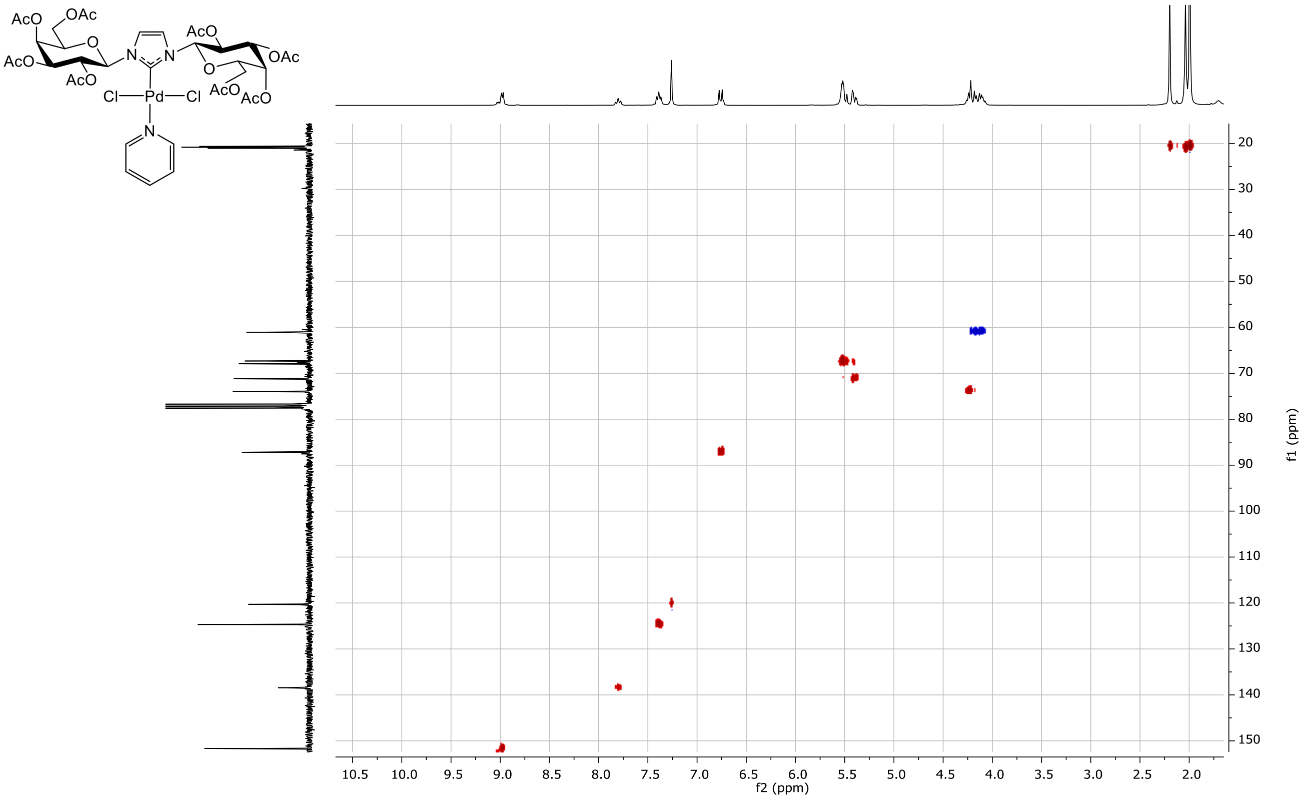

1-(2,3,4,6-Tetra-*O*-acetyl-β-D-galactopyranosyl)-3-(2,3,4,6-tetra-*O*-acetyl-β-D-galactopyranosyl) imidazol-2-ylidene palladium(II) pyridine dichloro (**3c**, FTIR-neat)

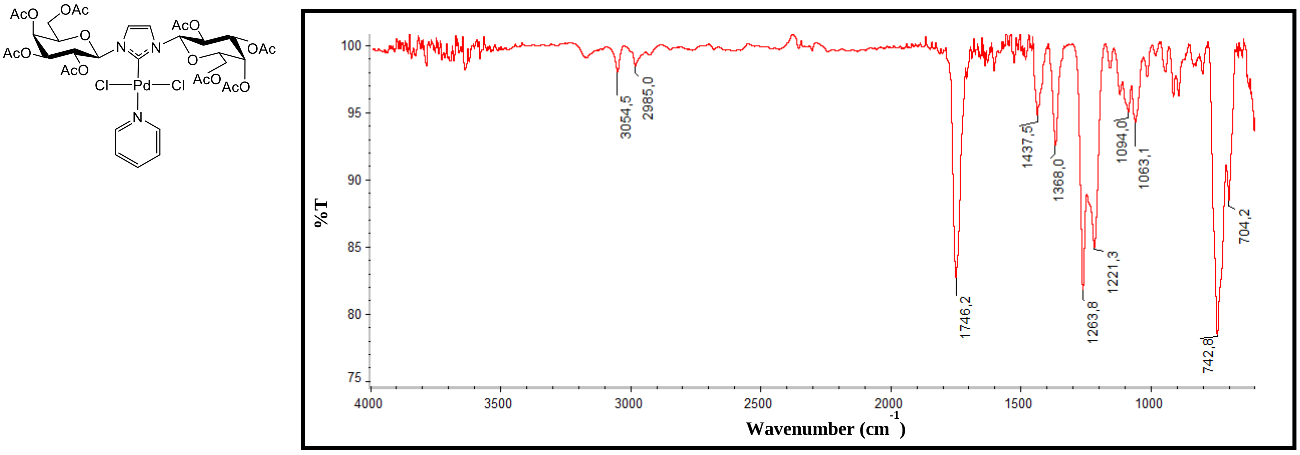

1-Butyl-3-(β-D-galactopyranosyl)imidazol-2-ylidene palladium(II) pyridine dichloro (**4a**, ^{1}H NMR, $CDCl_3$)

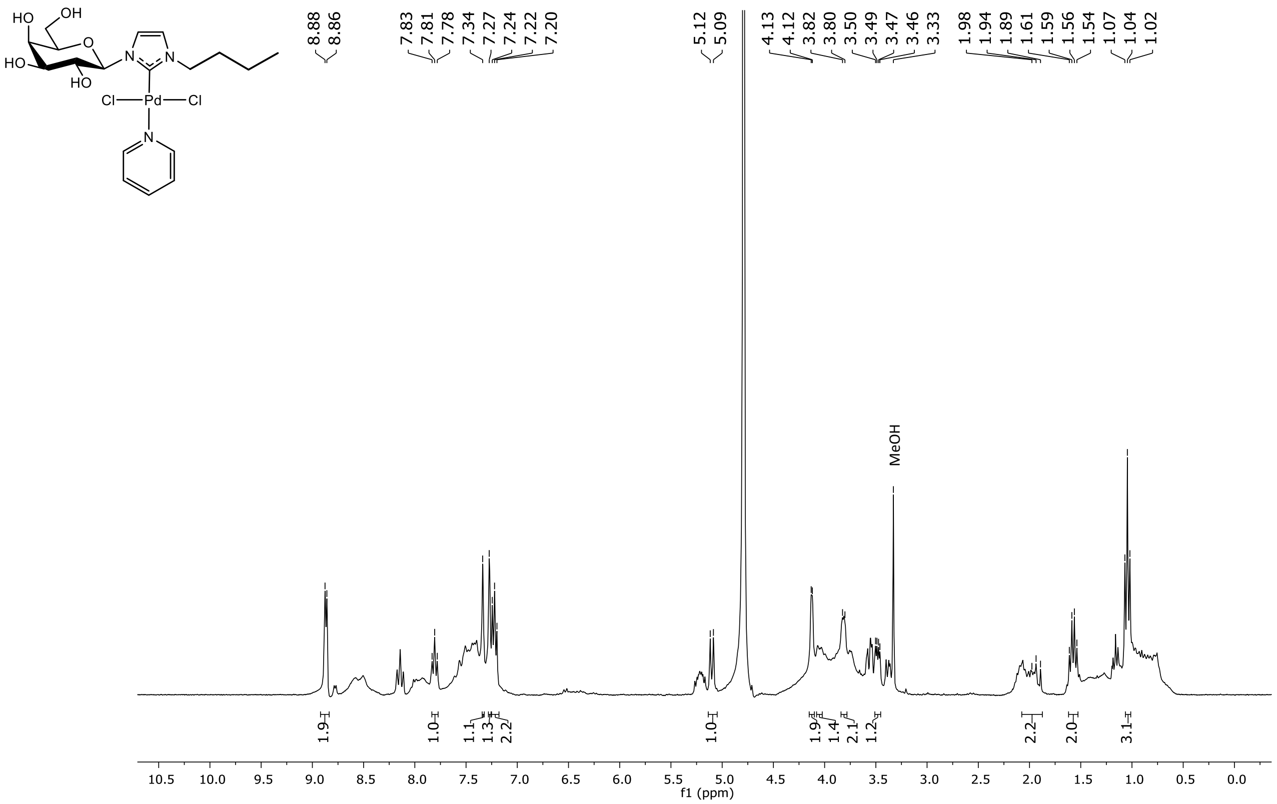

1-Butyl-3-(β-D-galactopyranosyl)imidazol-2-ylidene palladium(II) pyridine dichloro (**4a**, ^{13}C NMR, $CDCl_3$)

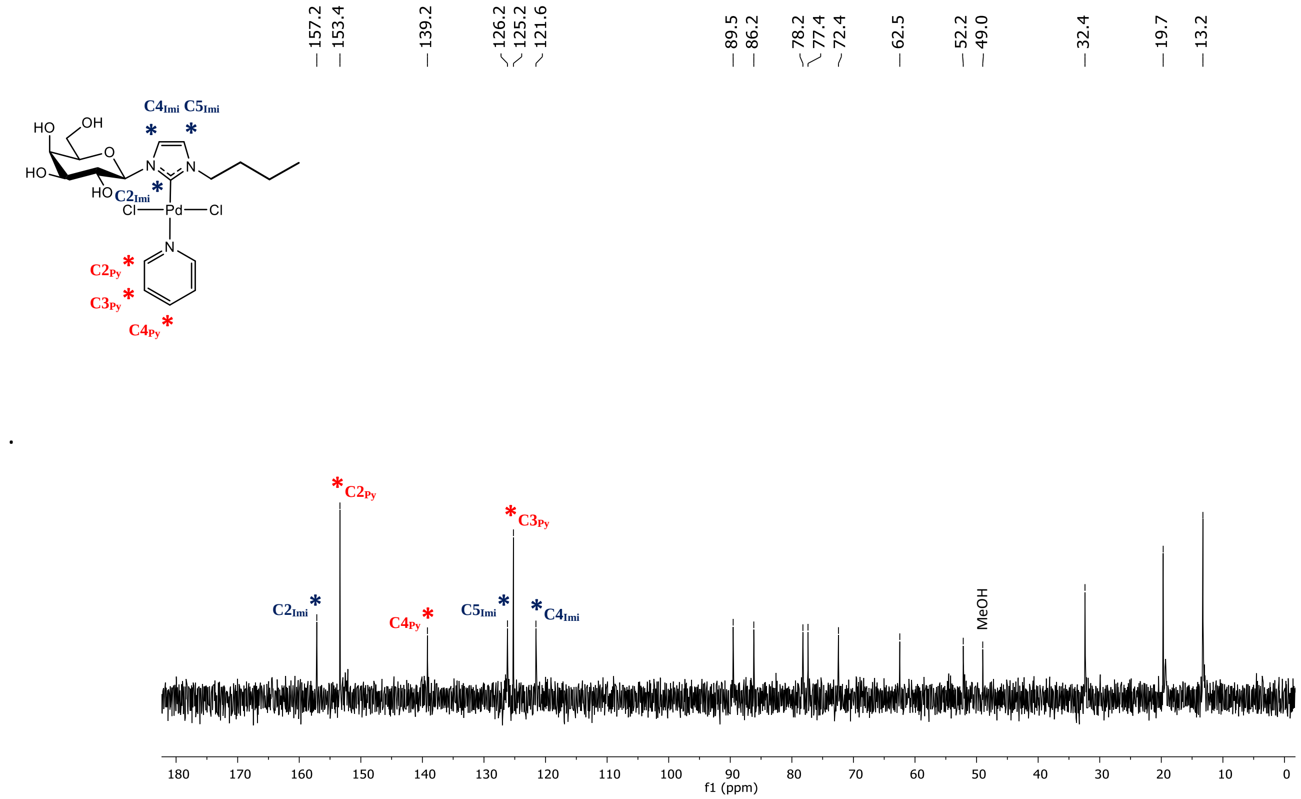

1-Butyl-3-(β-D-galactopyranosyl)imidazol-2-ylidene palladium(II) pyridine dichloro (**4a**, HSQC, $CDCl_3$)

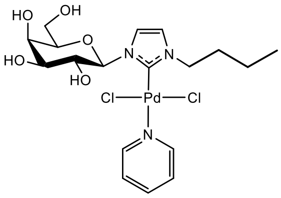

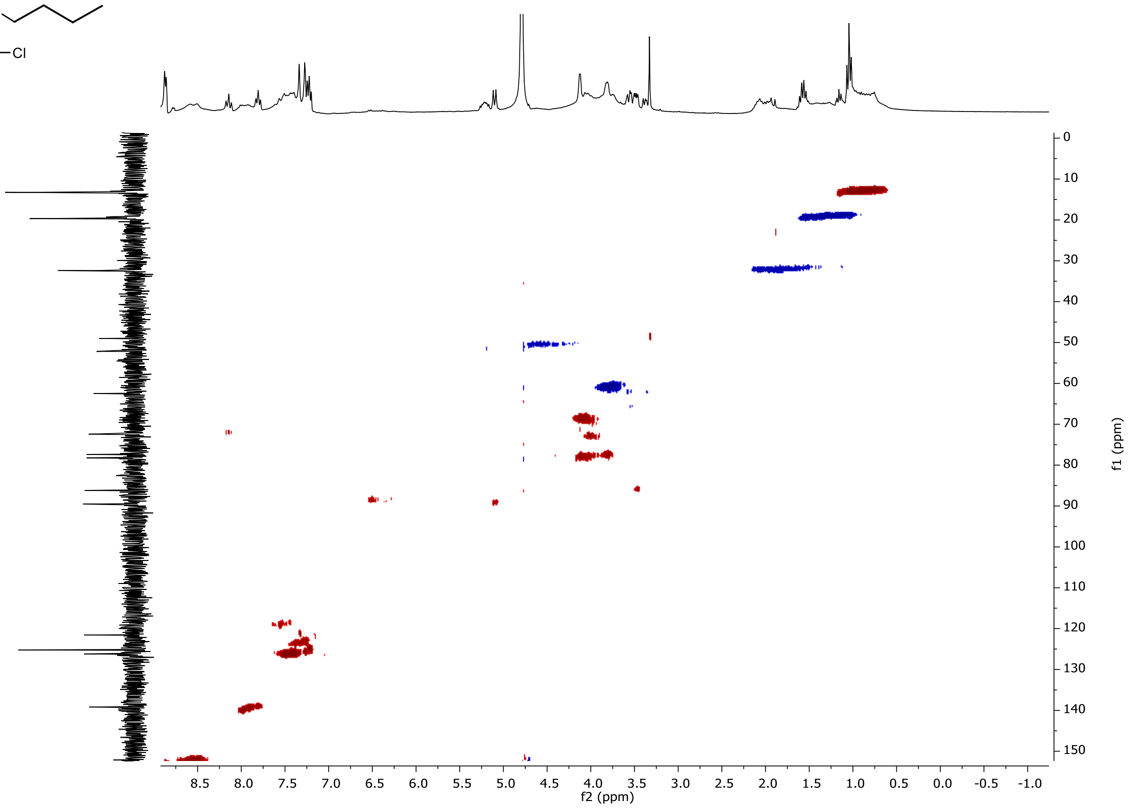

***o*-methoxybiphenyl** (^{1}H NMR, $CDCl_3$)

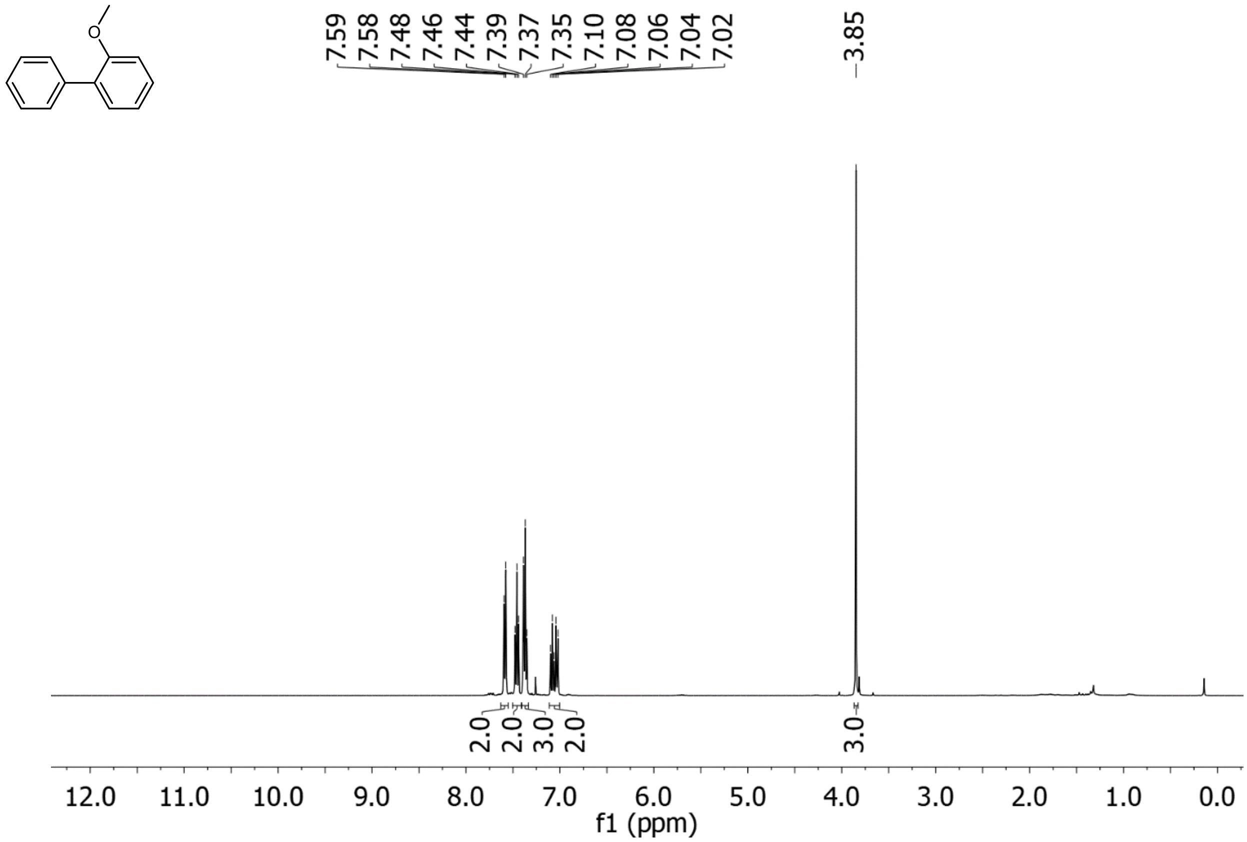

***o*-methoxybiphenyl** (^{13}C NMR, $CDCl_3$)

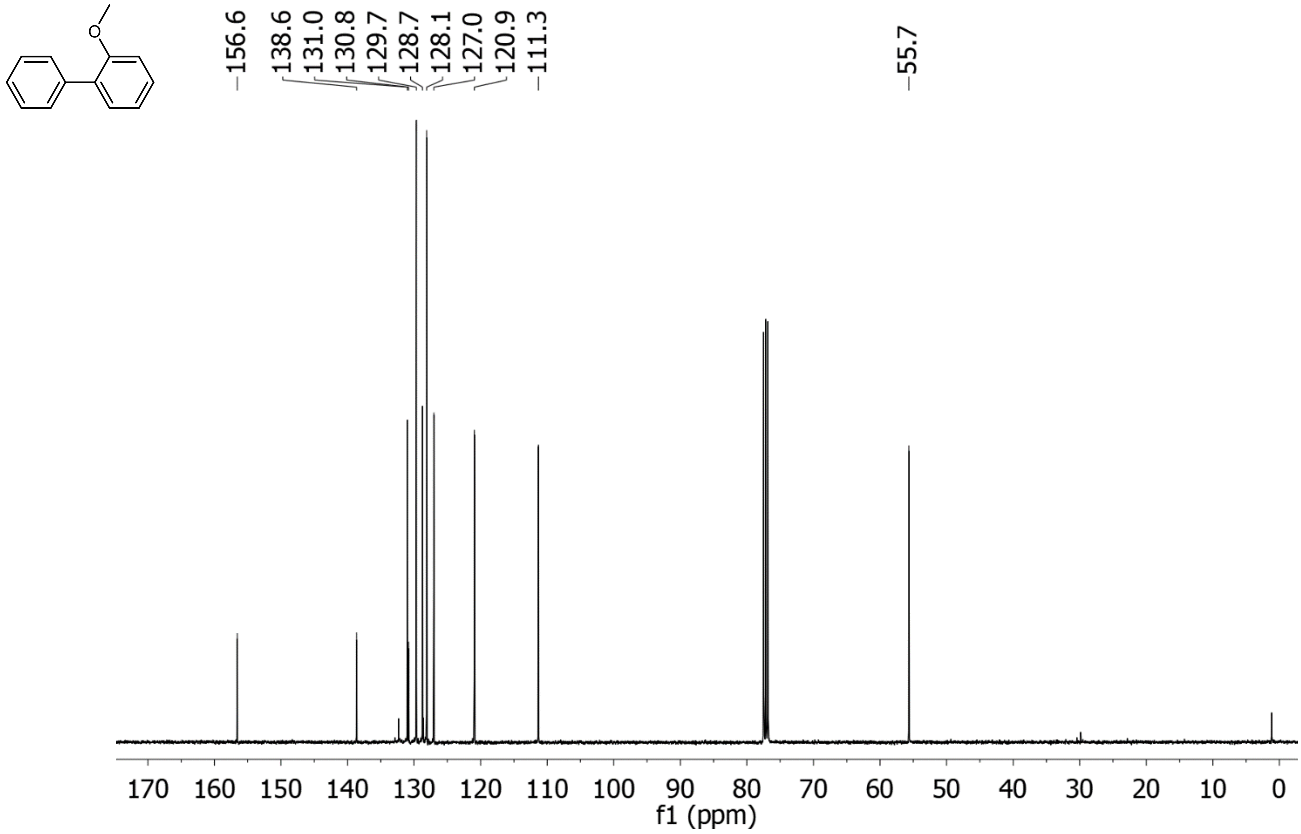

***m*-methoxybiphenyl** (^{1}H NMR, $CDCl_3$)

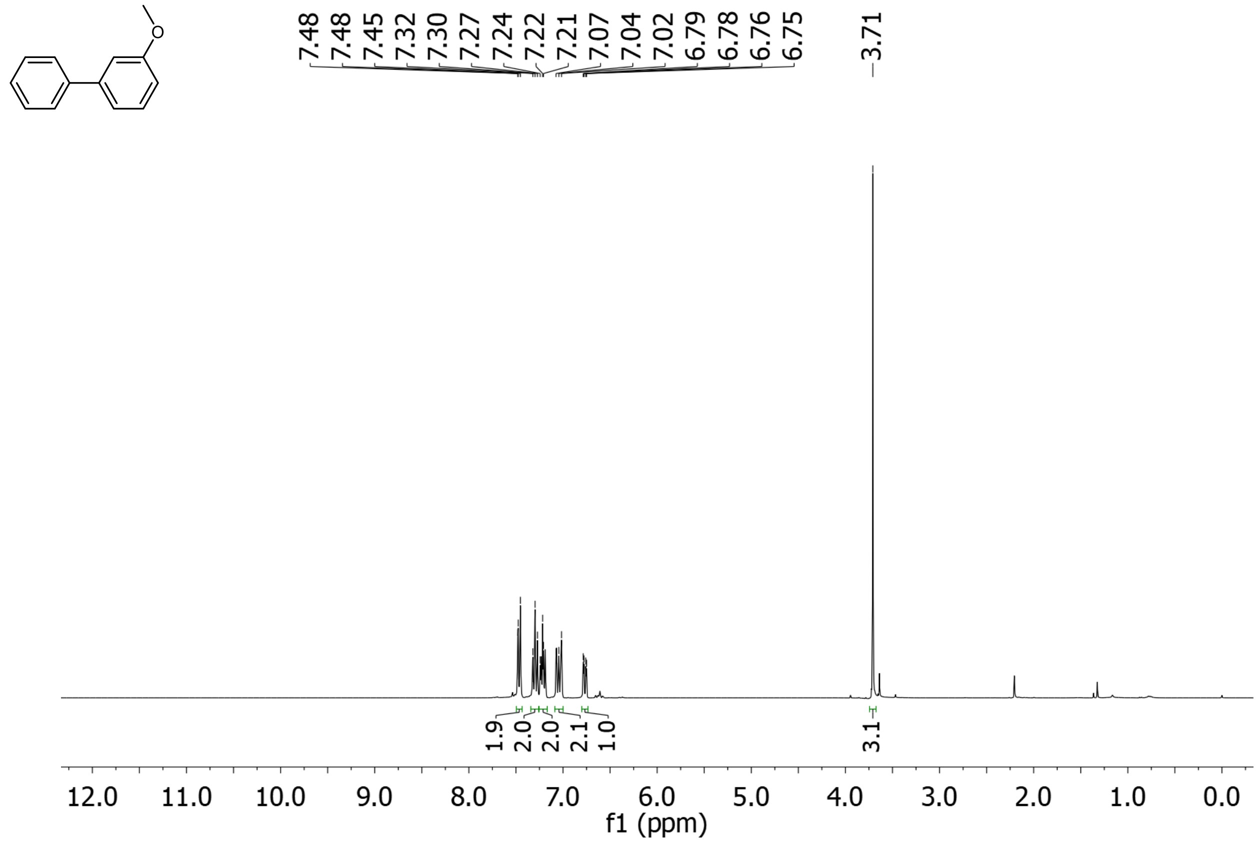

***m*-methoxybiphenyl** (^{13}C NMR, $CDCl_3$)

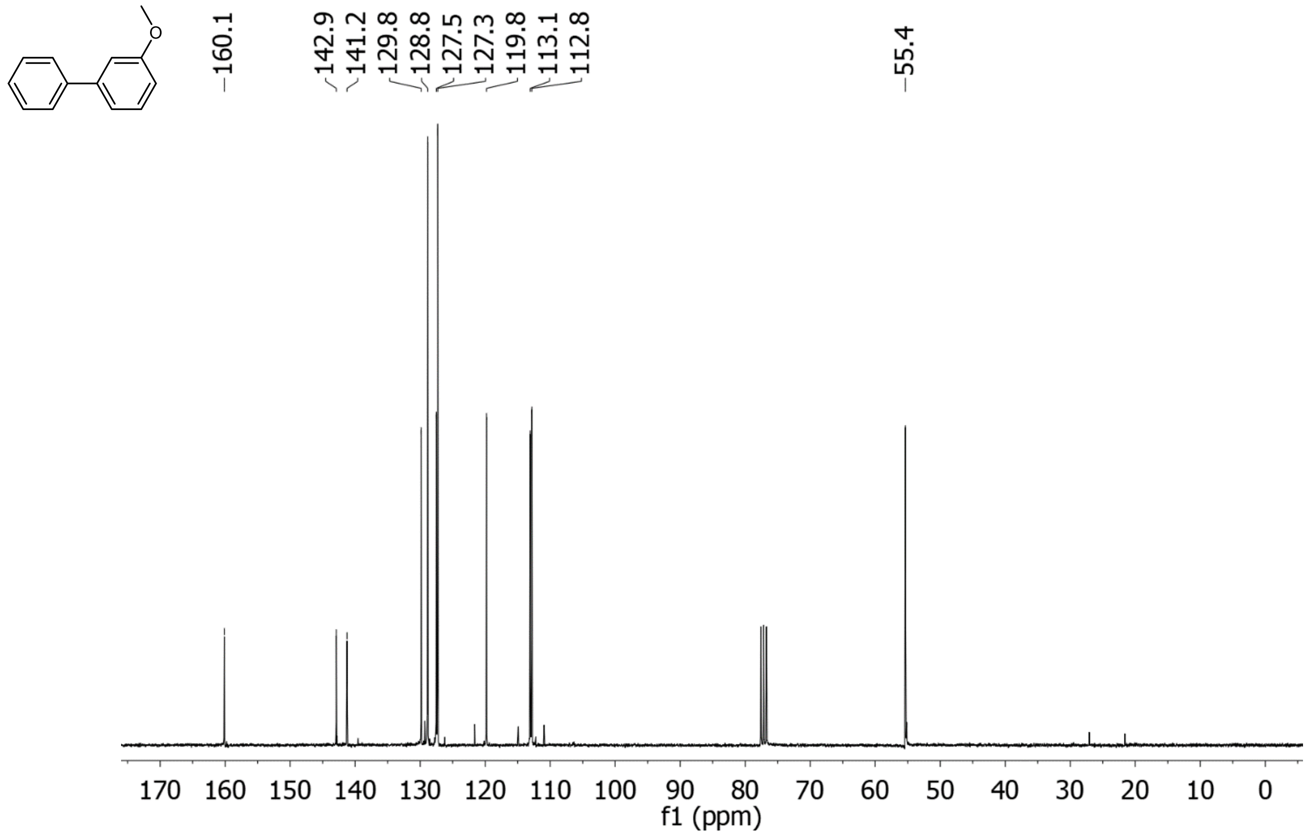

***p*-methoxybiphenyl** (^{1}H NMR, $CDCl_3$)

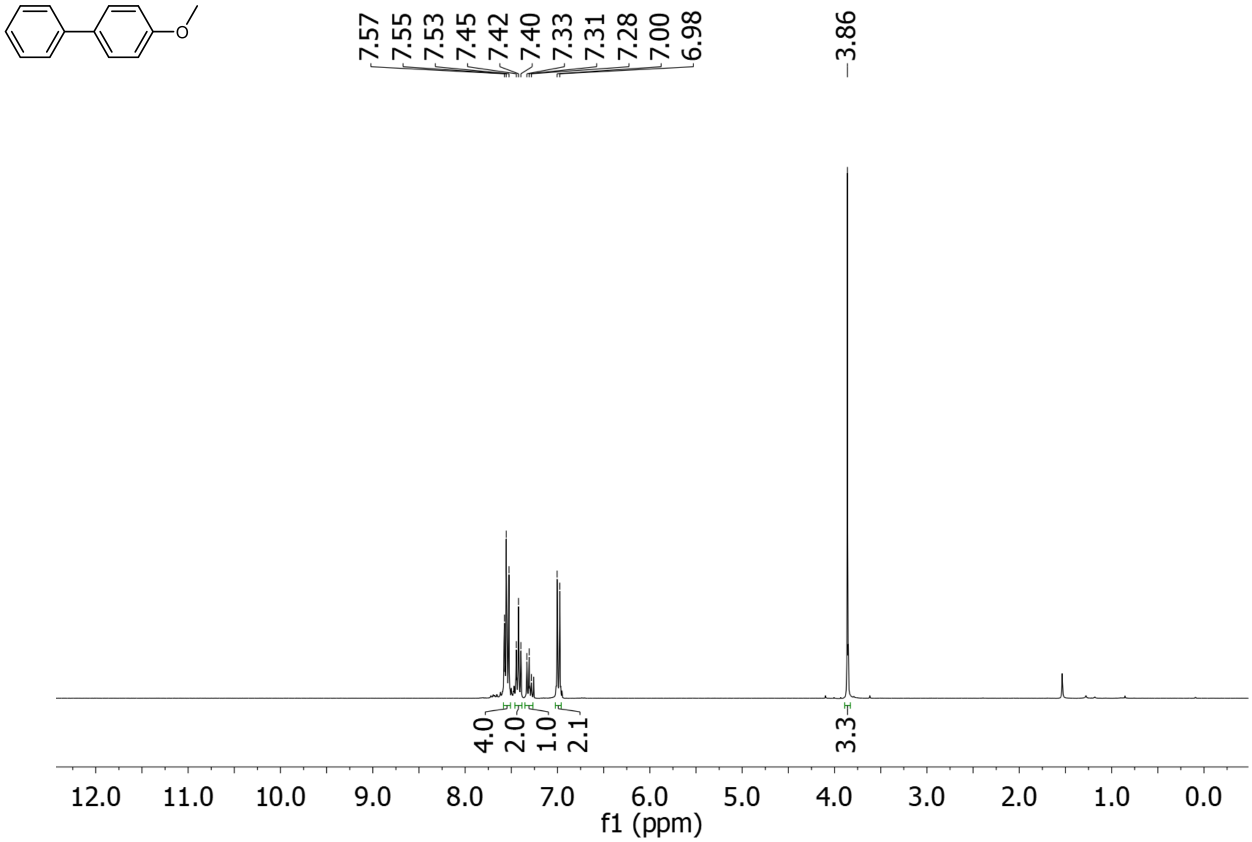

***p*-methoxybiphenyl** (^{13}C NMR, $CDCl_3$)

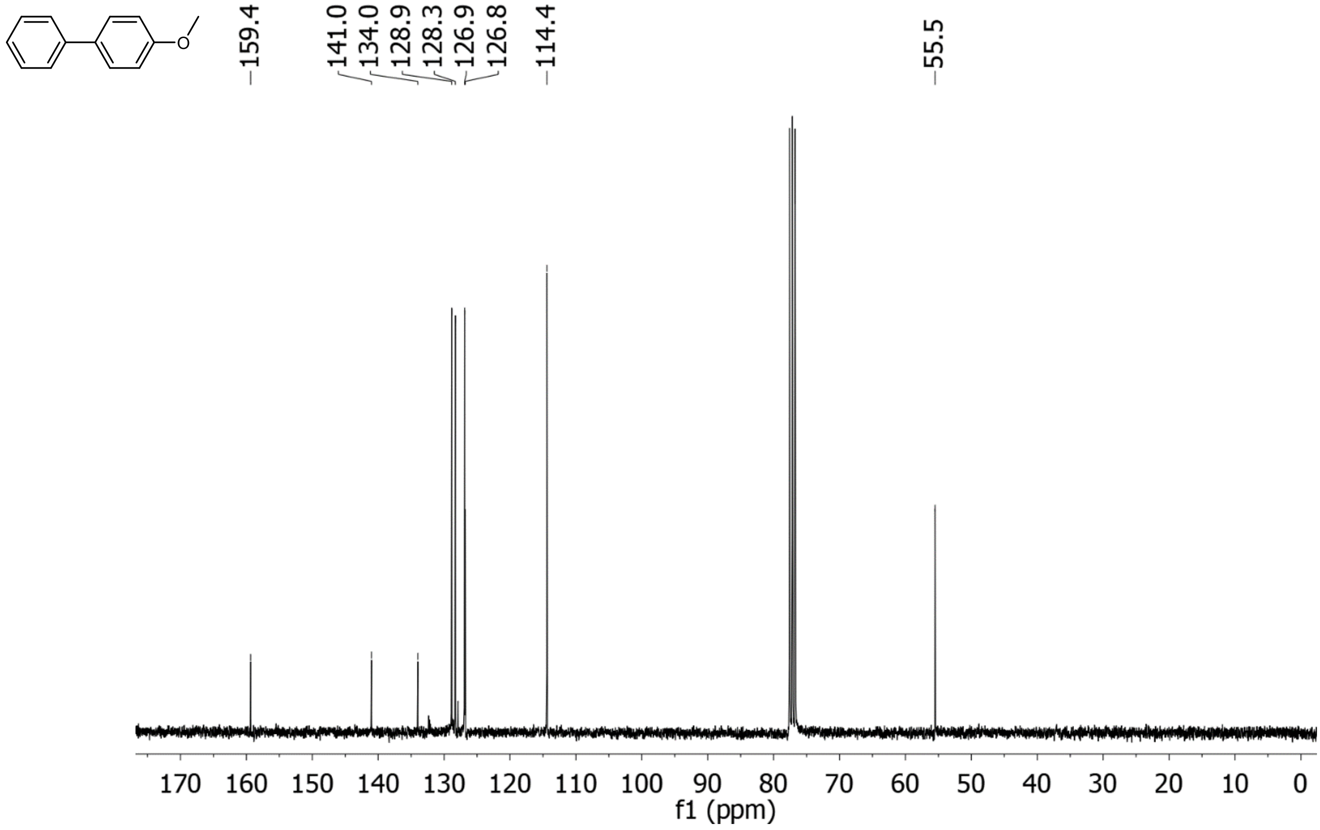

***m*-methylbiphenyl** (^{1}H NMR, $CDCl_3$)

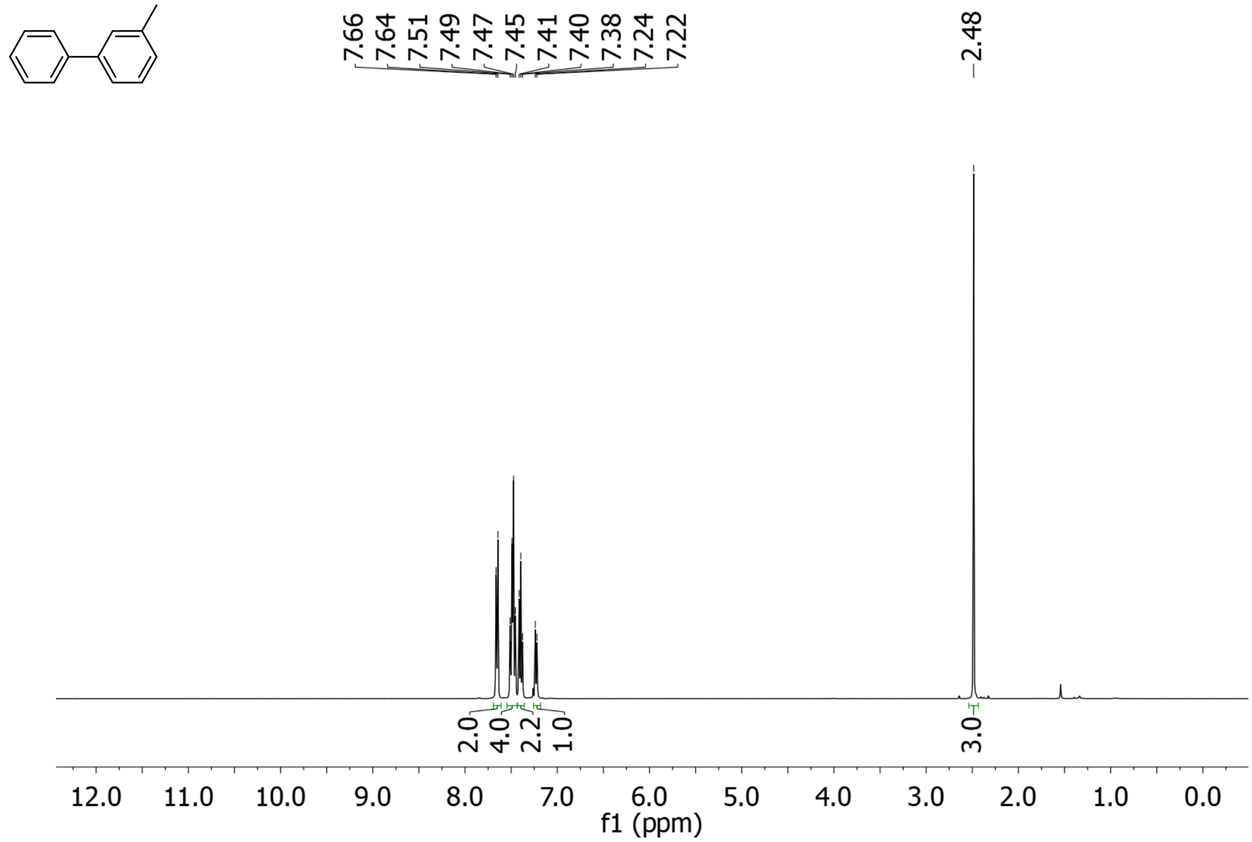

***m*-methylbiphenyl** (^{13}C NMR, $CDCl_3$)

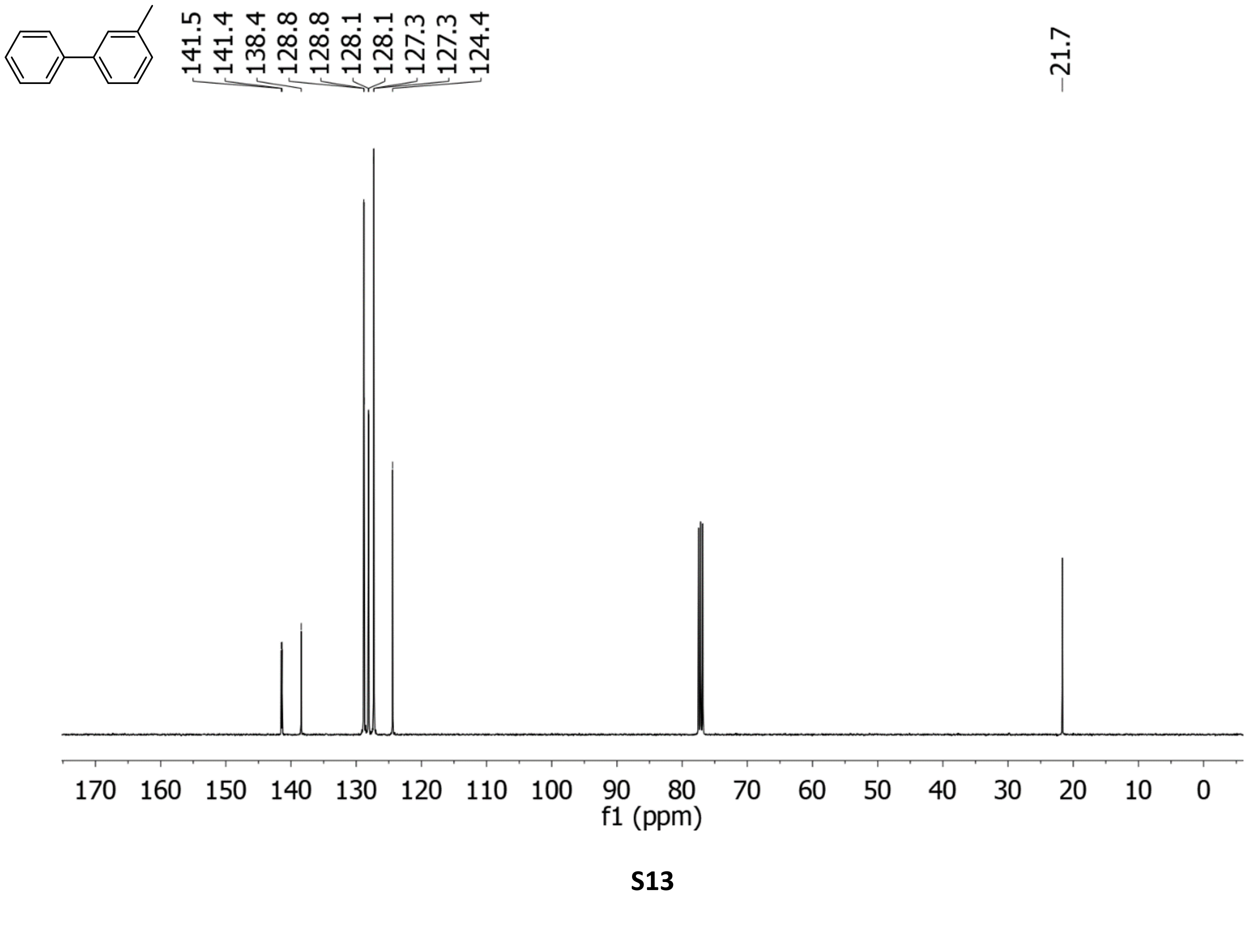

***p*-methylbiphenyl** (^{1}H NMR, $CDCl_3$)

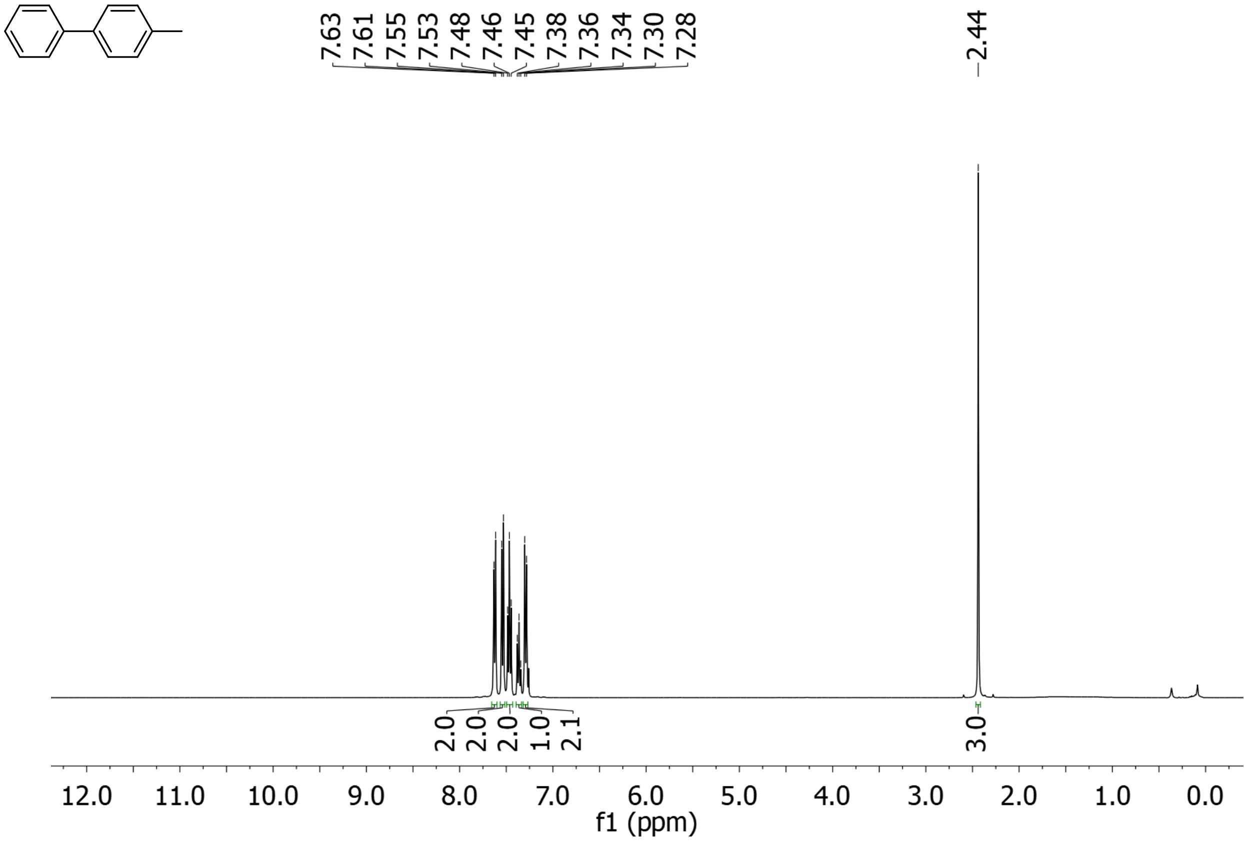

***p*-methylbiphenyl** (^{13}C NMR, $CDCl_3$)

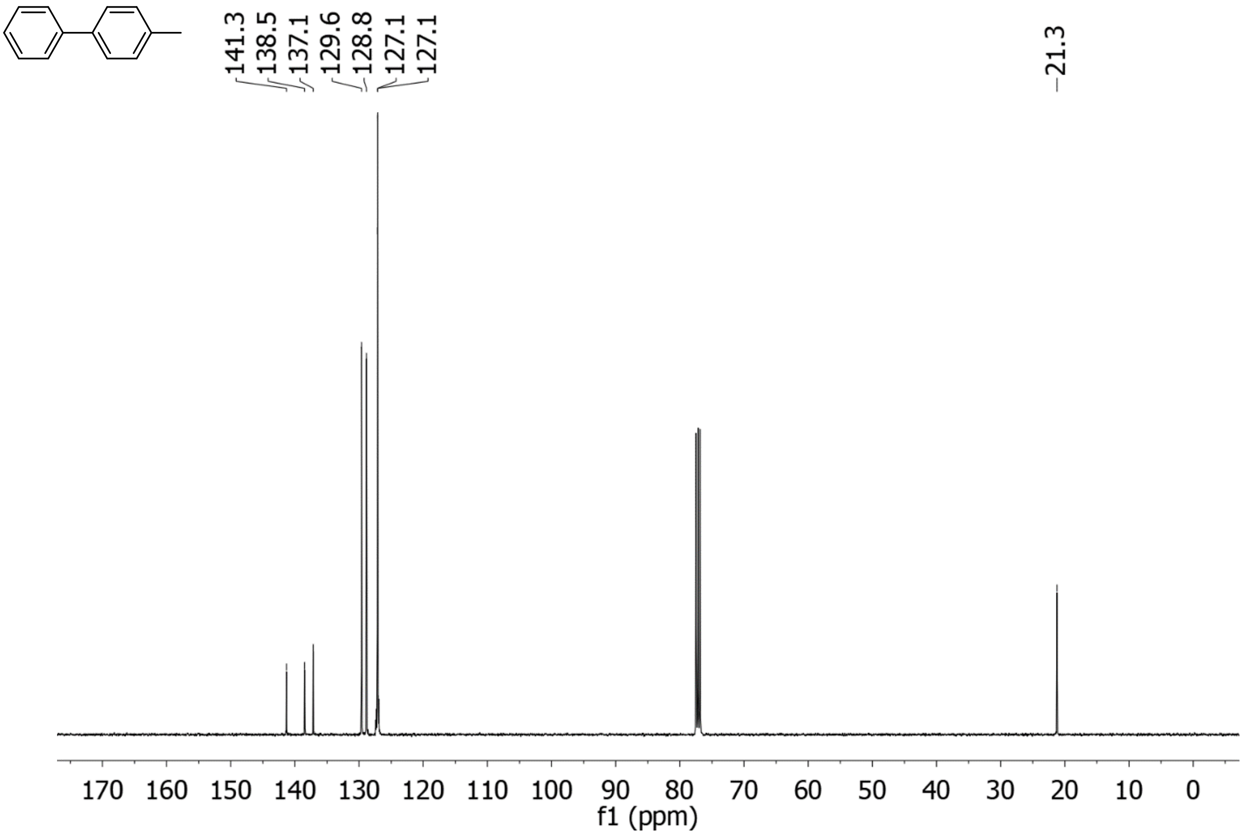

***m*-chlorobiphenyl** (^{1}H NMR, $CDCl_3$)

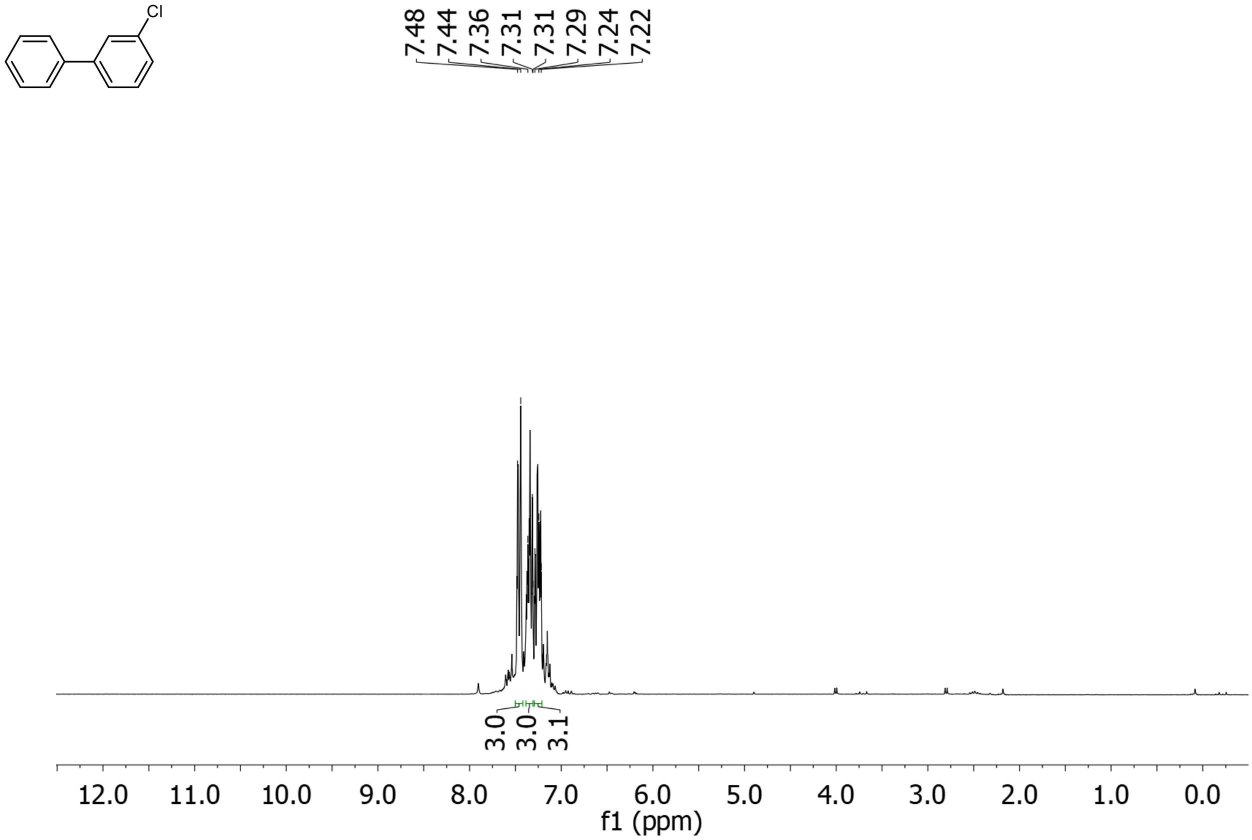

***m*-chlorobiphenyl** (^{13}C NMR, $CDCl_3$)

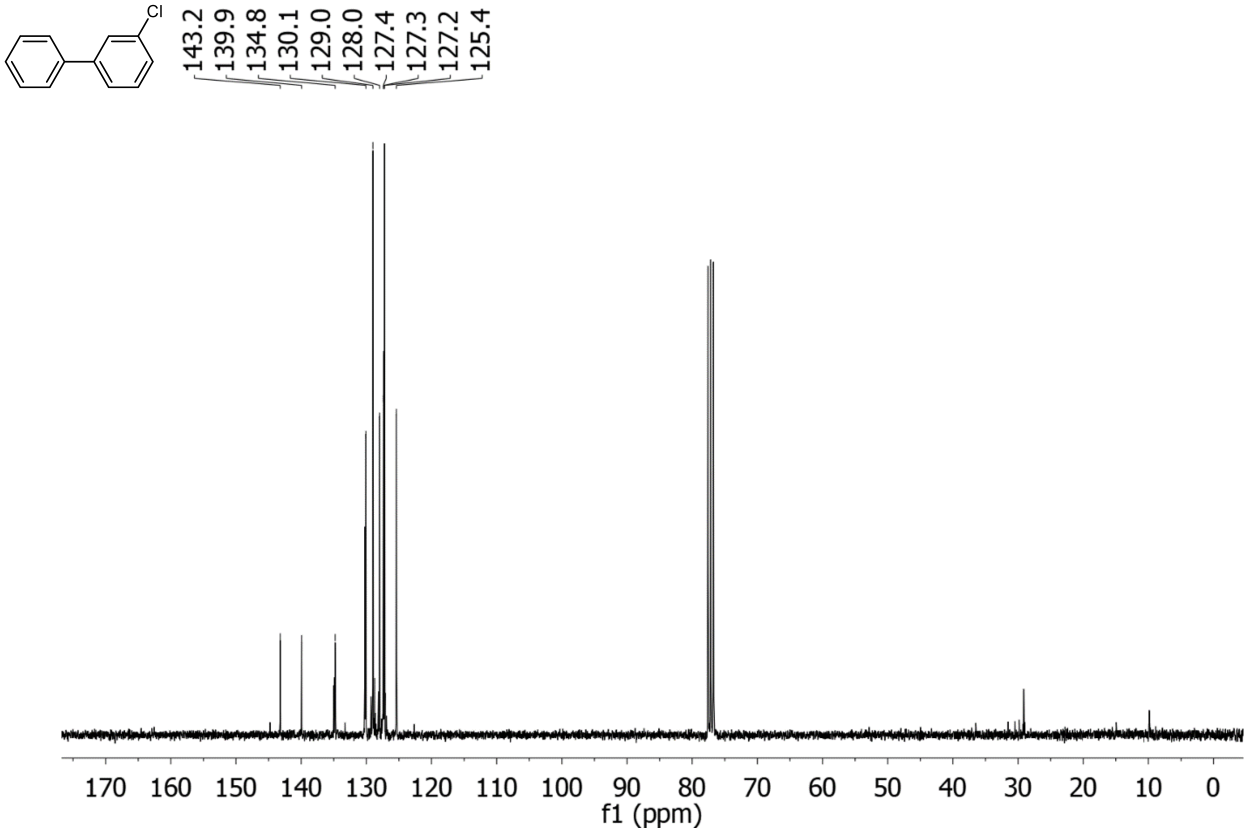

***p*-cyanobiphenyl** (^{1}H NMR, $CDCl_3$)

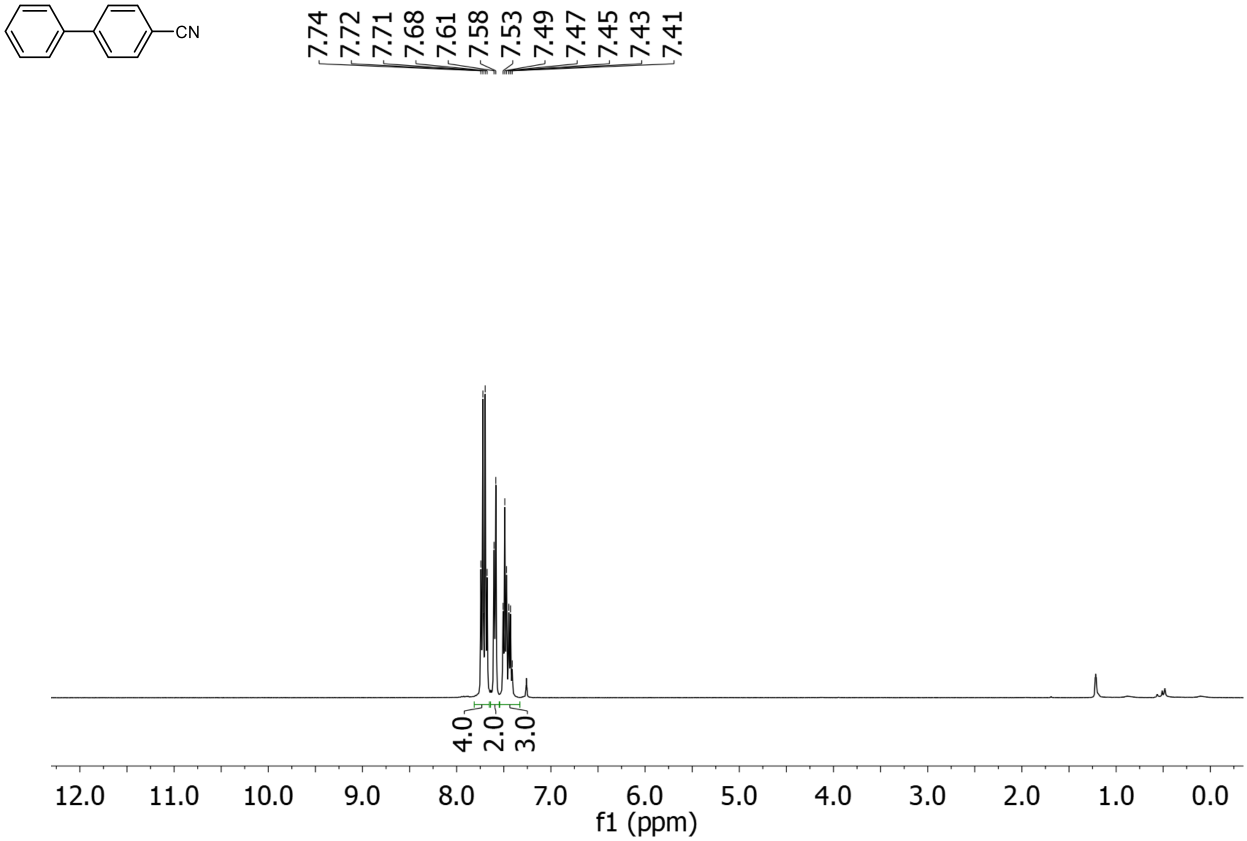

***p*-cyanobiphenyl** (^{13}C NMR, $CDCl_3$)

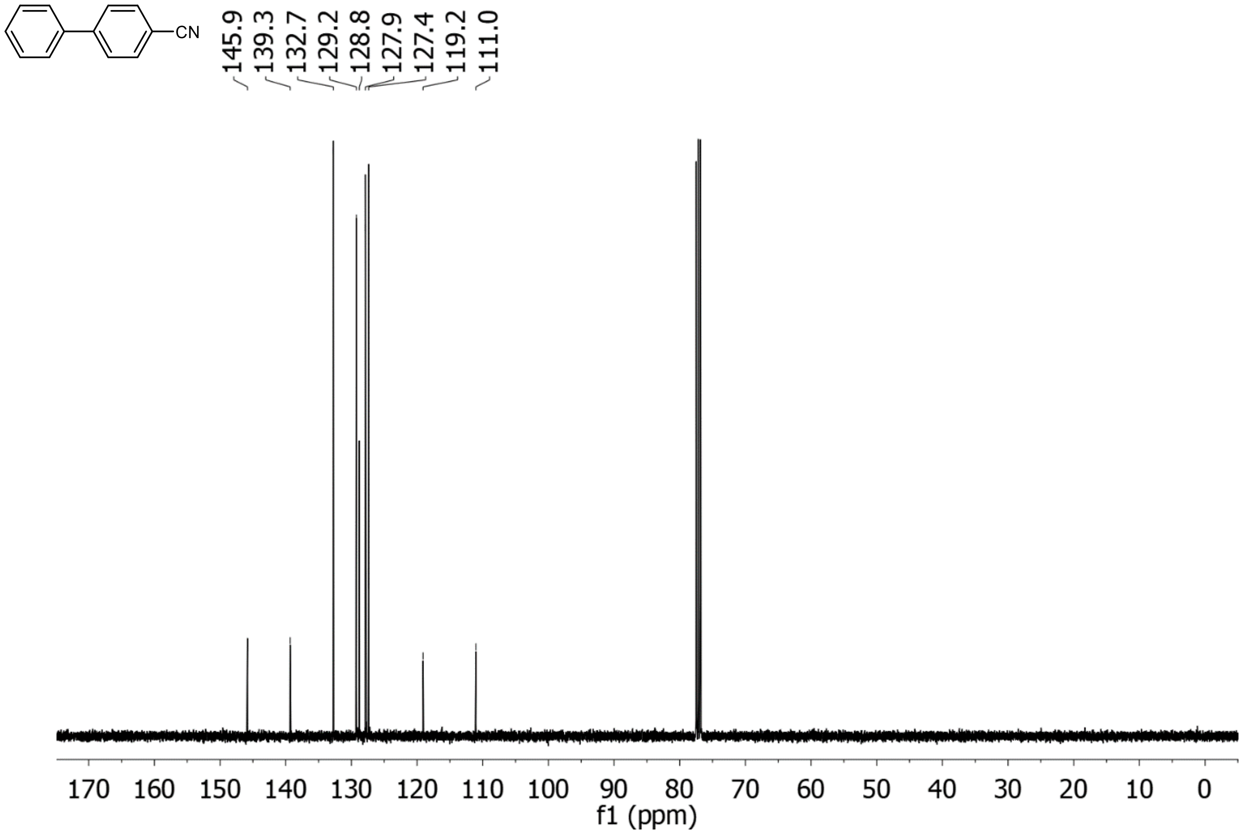

Supplement: RA-014-D3RA08031E-s001 [file RA-014-D3RA08031E-s001.pdf]
